# Opposing objective and subjective wellbeing outcomes within an environmentally vulnerable delta: a case study of Volta Delta, Ghana

**DOI:** 10.3389/fpsyg.2024.1401480

**Published:** 2024-08-23

**Authors:** Laurence Cannings, Craig W. Hutton, Alessandro Sorichetta, Kristine Nilsen

**Affiliations:** ^1^School of Geography and Environmental Science, University of Southampton, Southampton, United Kingdom; ^2^Dipartimento di Scienze della Terra “A. Desio”, Università degli Studi di Milano, Milan, Italy; ^3^Department of Social Statistics and Demography and WorldPop, University of Southampton, Southampton, United Kingdom

**Keywords:** objective wellbeing, subjective wellbeing, climate, hazards, landscape, agriculture

## Abstract

**Introduction:**

Despite a growing interest in the measurement and conceptualization of wellbeing, the integration within sustainability research, and the understanding of how different wellbeing outcomes relate, is limited. Many studies focus on singular, often objectively measured, outcomes, without acknowledging the breadth of available measures. This approach can result in crucial subjective information, which can be explored to understand actors’ behaviors and responses, being omitted from research and policy. This study explores objective and subjective wellbeing outcomes, and how they relate, within an environmentally vulnerable context. Wellbeing and environmental services are intrinsically interlinked, therefore, appropriate policy solutions are required to address human needs and pressures on supporting ecosystems.

**Methods:**

This paper uses binary logistic regression modelling, and qualitative participatory rural appraisal methods, to understand the environmental conditions, including climatic hazards and landscape characteristics, associated with households experiencing different objective/subjective wellbeing outcomes within Volta Delta, Ghana.

**Results:**

The mixed method approach highlights a differing relationship between inland agricultural areas impacted by drought and erosion, and coastal/riverine, peri-urban landscapes exposed to flooding and salinization. Agricultural areas associate with “poor but happy” outcomes, whereas peri-urban landscapes associate with being “non-poor but unhappy.” Drawing on existing literature, and both quantitative and qualitative results, these varying outcomes are hypothesized to be driven by differences in livelihood vulnerability, relative comparisons to others, responses to climatic hazards, and individualistic/collective wellbeing conceptualizations.

**Discussion:**

Our study concludes that environmental conditions influence objective and subjective wellbeing through different mechanisms. Sustainable development research should incorporate both objective and subjective measures when implementing and monitoring policy to more comprehensibly capture, and improve, wellbeing in environmentally vulnerable locations.

## 1 Introduction

Wellbeing and environmental services are intrinsically interlinked (Schleicher et al., 2018), therefore, appropriate policy solutions are required to address both human needs and pressures on supporting ecosystems. To achieve this, sustainable development research needs to contribute towards understanding wellbeing beyond traditional material and monetary approaches (Helne and Hirvilammi, 2015). However, despite developments in wellbeing measurement and conceptualization due to its growing importance as a policy outcome (Osei-Tutu et al., 2020), the integration of wellbeing research, and the understanding of how different wellbeing outcomes relate, within the field of sustainability is limited (Voukelatou et al., 2021). Consequently, a more comprehensive understanding of wellbeing within low-middle income countries (LMICs) is required (O'Mahony, 2022).

Wellbeing is broadly understood as a “favorable state of life desirable for every human being in the world at all times” (Böhnke and Kohler, 2010, p. 5). However, wellbeing is conceptualized in numerous ways across different disciplines (White, 2016). For example, objective approaches are primarily used in economics and development studies (Eid and Larsen, 2008), and subjective approaches in psychology (Cooke et al., 2016). Nevertheless, due to the various perspectives and methodologies, there is no requirement for a consensus on wellbeing measurement. Instead, multidisciplinary approaches that draw on different ideas are favourable to overcome each measurement’s limitations (Addison et al., 2008). Furthermore, White (2016) argues the absence of a consensus positively allows wellbeing to be discussed freely within communities, ensuring local needs are addressed.

This paper explores how environmental conditions associate with unaligned objective (OWB) and subjective wellbeing (SWB); measured through expenditure poverty and life domains unhappiness. Environmental conditions incorporate climate hazards, such as droughts and floods, and landscape characteristics, such as landcover classifications which implicitly contain information on livelihoods, ecosystem services, and social norms (Forkuo et al., 2021). Through the use of two binary logistic regression models, and qualitative discussions, the paper addresses the following research questions: (i) what is the spatial distribution of varying OWB/SWB outcomes across Volta Delta? (ii) how do climate hazards and landscape characteristics influence the relationship between OWB and SWB? (iii) what are communities’ and decision-makers’ environmental experiences, and how do they interact with wellbeing outcomes?

The study focuses on the role of environmental conditions due to deltas being defined as “one of the most vulnerable environments threatened by climate and human-induced changes” (Foufoula-Georgiou et al., 2013, p. 4). Key documented climate hazards in global deltas include; flooding, coastal erosion, subsidence, soil and water salinization, storm surges and cyclones (Ericson et al., 2006; Syvitski, 2008; Kuenzer and Renaud, 2012). The broader suite of potential wellbeing determinants, such as employment status and social relationships (Osei-Tutu et al., 2020), are interpreted to function within the broader context of environmental vulnerability, and therefore will be discussed through an environmental lens.

Due to the combination of high population densities (Ericson et al., 2006), economic potential, environmental vulnerability, and dependency upon ecosystem services for human and material wellbeing, deltas, many of which are located in LMICs, are often targeted by international development (Foufoula-Georgiou et al., 2013). Analysis exploring OWB and SWB is not exclusive to deltaic communities; however, due to the multiple challenges experienced, these locations provide opportunities to research the different mechanisms and processes which influence the numerous components of wellbeing (White, 2016). Wellbeing is “relational,” meaning it is constructed within a specific time and space (Gough and McGregor, 2007), therefore similar analysis should be replicated in different locations to further understand how local factors influence wellbeing outcomes.

Addressing the research questions, and exploring complex relationships between OWB and SWB within an environmentally vulnerable context, is justified by the limited empirical evidence linking wellbeing and environment (Schleicher et al., 2018). Furthermore, traditional environment-wellbeing studies prominently focus on singular outcomes, implicitly assuming singular measures capture the totality of wellbeing (Barbier and Hochard, 2018; Nanor et al., 2021). In particular there is an underutilization of SWB in LMIC studies, with much research, especially within the context of global environmental challenges, located in high-income countries (HICs) (Sulemana et al., 2016). Limited focus within LMICs could be attributed to Maslow’s “hierarchy of needs” framework, with poorer countries hypothesized to focus on immediate physical needs, rather than “bonus” psychological needs encompassed within SWB (Chumo et al., 2023).

Nevertheless, Flik and van Praag (1991) state “individuals themselves are the best judges of their own situation” (p. 313); therefore, SWB measures can minimize the influence of external assumptions and provide more accurate, context-specific results (Nunan, 2015). For example, while utility theory economics suggests higher financial capital increases happiness by enabling individuals to satisfy more of their preferences through market transactions (White, 2016), research has indicated that within certain LMIC communities, access to education and health services are often prioritized over private incomes due to limited public funding, and the immediate threats to survival from climatic, social and health shocks (Dowling and Yap, 2012).

Individuals’ emotions and perceptions, incorporated within SWB, also govern behavior (Alam and Mallick, 2022). Therefore, exploring SWB within sustainability research can enhance decision-makers’ understanding of communities’ priorities and actions, particularly if they do not align with targeted objective outcomes, thereby improving buy-in and policy success. For example, Gross-Camp (2017) found local support in Tanzanian community-based forest management projects to be high, despite increased financial inequality, due to the pride and heightened SWB derived from engaging with external organizations and actively conserving their environment.

Focusing on opposing OWB/SWB outcomes is pivotal in supporting decisions to include various wellbeing measures when implementing and monitoring policy. This research approach can help illustrate to policymakers the dangers of narrowly prioritizing specific outcomes at the expense of other wellbeing components (Copestake, 2008). For example, research deploying an exclusively “objective” approach may omit information on how collective community norms could promote sustainable activities (Bouma et al., 2008), whereas a “subjective” approach may overlook requirements for more-immediate material support (Davis, 2014).

Limited studies look at how OWB and SWB relate in LMICs, with past research explicitly comparing these outcomes (i.e., Mahmood et al., 2019) often not focusing on how environmental conditions influence the association. Viewing this relationship through an environmental lens ensures the study acknowledges the interconnectivity between environmental conditions and livelihoods, food security, service access and social identities in LMICs (Cazcarro et al., 2018; Addo et al., 2018a). Existing studies produce varying results regarding whether OWB and SWB align or oppose in different contexts (Böhnke and Kohler, 2010), with the main narrative being that OWB and SWB are related, yet not interchangeable (Meisenberg and Woodley, 2015; Cannings et al., 2024). The potential for different mechanisms to influence OWB and SWB justifies this study’s focus on how environmental conditions may contribute differently towards outcomes from diverse disciplines.

## 2 Background

### 2.1 What is objective and subjective wellbeing?

OWB incorporates universal, measurable components relating to quality of life which are given meaning by socially constructed desirable norms (Vaznonienė, 2014), such as income or educational attainment (Western and Tomaszewski, 2016). Financial OWB for example uses a neoclassical approach, which is positioned within “utility theory,” where monetary capital is interpreted as a universal right, permitting individuals to act freely in a capitalist society and satisfy their needs by consuming services and goods (Rasool et al., 2011). OWB measures are prominent within sustainable development, highlighted by all UN Sustainable Development Goals (SDGs) being objectively monitored. For instance, SDG1 measures the proportion of people with <$1.25/day (UN, 2022).

In contrast, SWB captures individuals’ internal assessments, cognitive judgments and affective reactions to their life and environment (Stone and Mackie, 2013). Subjective outcomes are developed from “affects,” consisting of emotions and moods, and “thoughts,” referring to individuals’ perception of their lives in relation to others, past time periods, and socially constructed aspirations (Veenhoven, 2009; Vaznonienė, 2014). According to Dolan et al. (2011), SWB sits within three broad categories: (i) “evaluation”; general assessments of life (ii) “experience”; balance of positive/negative emotions (iii) “eudaimonic”; inherent, universal psychological positives. Different theories exist regarding eudaimonic SWB; for example, Jax et al. (2018) suggest “caring for nature” is an absolute positive, whereas Deci and Ryan (2012) note three needs of autonomy, relatedness (social connection) and competence (mastery of skills/environment); the latter two are particularly important within collective agricultural communities (Markussen et al., 2018).

Quantifying the concept of “happiness” from an “evaluation” perspective is the most common approach in social science (Dolan et al., 2011). However, quantifying complex social phenomena brings challenges; for example, static statistical outputs juxtapose the fluidity of individual and societal processes, and broad quantifiable relationships potentially generalize personal experiences (Shaffer, 2013). Nevertheless, the importance of parsimony and pragmatism from a policy perspective is recognized, with this paper aiming to illustrate the benefit of including SWB, alongside OWB, within sustainable development. An absence of a consensus when defining or measuring “happiness” should not prevent action, with recent improvements in the available methodological tools providing easy to understand, actionable metrics for policymakers (Sachs, 2019). The ability to use quantifiable “happiness” measures to create practical initiatives is illustrated in UAE, which founded the Global Council for Happiness and Wellbeing “to identify best happiness practices of government, businesses, schools, city planning [and] health systems” (Sachs, 2019, p. 6). Furthermore, designing development policy through a “happiness” lens can ensure comparability and collaboration across different actors and departments (Helliwell, 2019), as despite discrepancies over its theoretical underpinnings, as a concept, “happiness” is universally relatable and recognized as innately positive (Dasgupta, 2022). Therefore, despite SWB being a multidimensional, personal outcome, calculating quantifiable metrics can facilitate its inclusion within spaces, such as sustainability and environmental justice studies, which are historically dominated by quantitative methods (Althor and Witt, 2020), and ensure research findings are practical and actionable.

### 2.2 Are objective and subjective wellbeing related?

**Figure 1:**
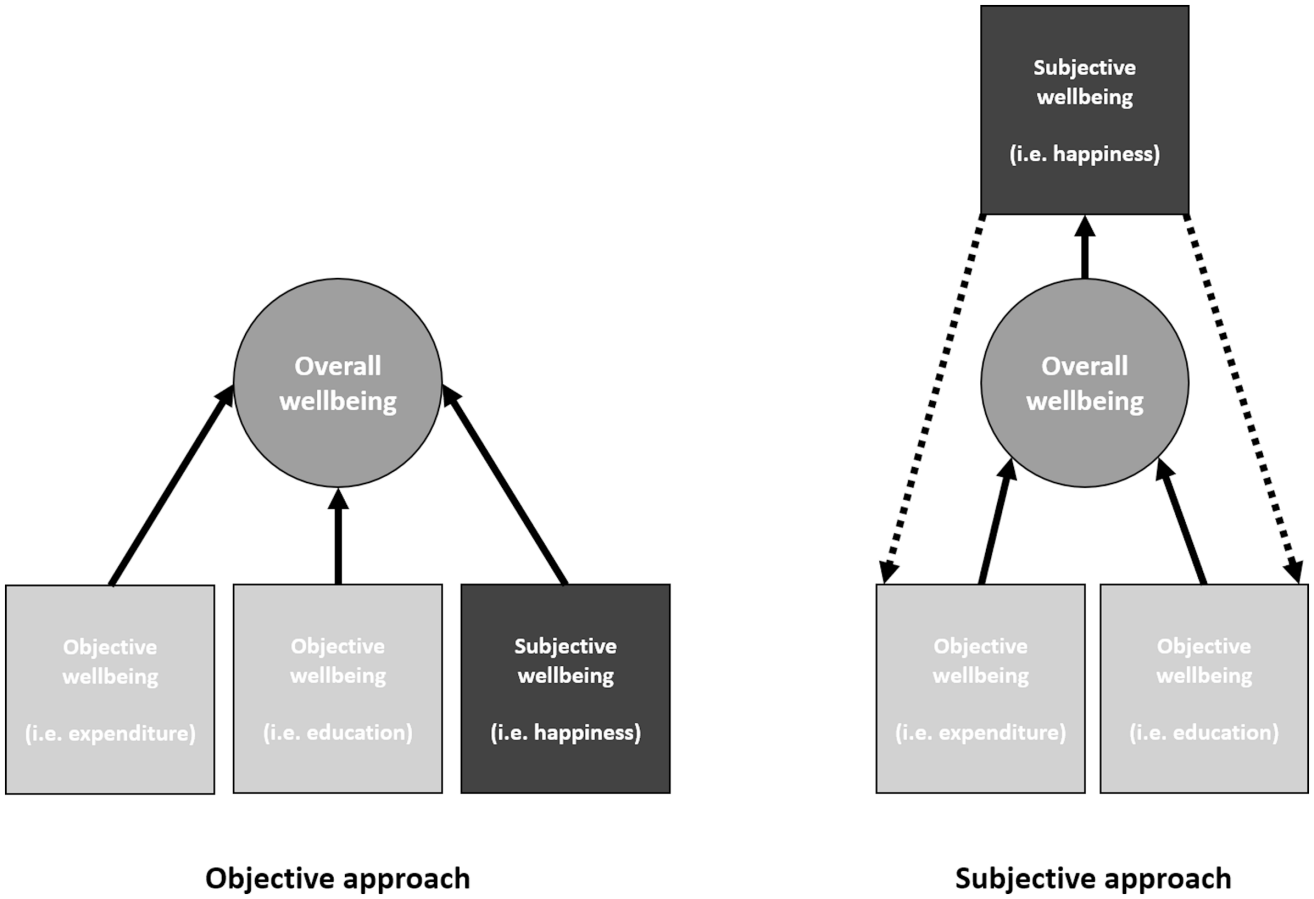
Adapted illustration of Böhnke and Kohler’s (2010) “objective” and “subjective” approaches to objective/subjective wellbeing associations.

When examining whether OWB and SWB align or oppose one another, Böhnke and Kohler (2010) present two interpretations (Figure 1). Firstly, an objective approach where SWB is independent of OWB, allowing for opposing OWB/SWB. Next, a subjective approach, where objective resources influence overall wellbeing, which is then reflected in SWB, suggesting OWB/SWB align. As wellbeing is “relational,” the relationship between OWB and SWB may differ across local contexts.

This paper investigates the “objective” approach, where OWB/SWB oppose one another (e.g., Bradshaw and Finch, 2003; Graham and Pettinato, 2006; Alem et al., 2014; Posel and Rogan, 2016). Firstly, drawing on Easterlin’s (1974) Paradox, individuals’ aspirations increase in relation to objective wealth, termed the “hedonic treadmill,” creating constantly repositioning gaps between desires and fulfilment. An inability to reach these goals can limit SWB (Zhang and Churchill, 2020), particularly within countries like Ghana which experienced rapid growth (Crawford and Abdulai, 2009). These ideas are supported by previous Ghanaian studies suggesting financial capital to not be relevant to a “good life” beyond avoiding absolute poverty (Dzokoto et al., 2019); “once subsistence income is guaranteed, making people happier is not easy” (Layard and Layard, 2011, p. 4).

Others argue financial OWB represents “potential,” rather than “realized” wellbeing (Sen, 2005; Gasper, 2007). Transforming “potential” to “realized” wellbeing requires skills, institutional support, and favorable environments. Without such resources individuals may be unable to translate financial OWB into SWB. For example, insufficient health infrastructure could lower health, regardless of objective wealth, and consequently lower happiness (Addai and Adjei, 2014).

The relationship between OWB and SWB may also differ spatially. For example, urban populations with greater media exposure and mobility may possess higher material aspirations, and place greater weight on relative positionality than remote rural communities when defining their SWB (Corazzini et al., 2011; Ng and Diener, 2014; Ravallion, 2014). In contrast, rural communities may achieve material needs through non-market channels, such as subsistence agriculture and community trading, or be content with lower levels of financial OWB which enable the fulfilment of non-material needs such as community relations, safety, and cultural identity (Ward and King, 2016). These ideas relate to the “happy farmer” identity (Crossley, 2012; Markussen et al., 2018), which suggests poorer, rural communities achieve higher SWB through place attachment (Albrecht et al., 2007) and “bonding” social capital; defined as “strong relationships that develop between people of similar background and interests [who] … provide material and emotional support” (Claridge, 2018, p. 2).

Low SWB can also be experienced across the OWB spectrum (Ravallion et al., 2013). For example, objectively non-poor households may encounter unhappiness if climatic hazards cause their current situation to be relatively worse-off than their neighbors or a previous point in time; particularly in unequal societies with differing capacities to adapt to changing conditions (Haughton and Khandker, 2009).

The capacity for OWB and SWB outcomes to oppose one another highlights the importance of incorporating broad elements of wellbeing within sustainable development policy. This shift in focus could broaden the scope of success for Ghanaian development projects, which have traditionally neglected local experiences and narrowly focused on top-down economic initiatives (Domfeh and Nyigmah Bawole, 2009). The incorporation of SWB targets could also be cost-effective; for instance, interventions focused on harnessing community togetherness are less capital and environmentally-intensive than income-generating initiatives (Dolan and White, 2007).

### 2.3 Environment and wellbeing

By exploring how environmental conditions associate with opposing OWB/SWB, this paper contributes towards understanding elements of the “environment-wellbeing nexus” (Szaboova et al., 2020). Environment-wellbeing relationships are complex and multidirectional, preventing causal relationships from being defined (Heger et al., 2018); opposing “downward spiral theory” which suggests an inevitable link between environmental stress and poverty (Reardon and Vosti, 1995). Whilst acknowledging potential multidirectional feedback effects, this paper specifically focuses on environmental impacts on wellbeing.

Drawing on the IPCC *risk* framework (Reisinger et al., 2020), LMICs are most vulnerable to climate hazards due to; (i) greater exposure (Brown et al., 2007) (ii) higher sensitivity due to the economic reliance upon threatened ecosystem services (Sedda et al., 2015) (iii) lower adaptive capacity due to insufficient human capital, credit access, information systems and agricultural technology (Asante and Amuakwa-Mensah, 2015). However, despite facing the gravest consequences of atmospheric warming on a global scale, these locations, such as West Africa, are minor contributors (Roberts, 2001). Althor et al. (2016) showed 30, predominantly tropical, countries to be within the bottom two quintiles for carbon emissions, yet the top two for climate change vulnerability (2010)^1^. Ghana is estimated to experience the highest vulnerability levels by 2030. This global disparity in exposure and vulnerability is hypothesized to be “an outcome of skewed development processes” (Cardona et al., 2012, p. 70), including neocolonial legacies, uneven investment patterns, natural resource mismanagement and limited livelihood options for the poor (Greiner and Sakdapolrak, 2016).

Environmental vulnerability is intertwined with both OWB and SWB (Thomas et al., 2019). For example, exposure to drought and soil erosion within sensitive agricultural landscapes can negatively influence financial OWB by causing crop failures (Cazcarro et al., 2018), livestock mortality (Serdeczny et al., 2017) and increasing regional food prices (Schneider and Gugerty, 2011). Similarly, exposure to flooding may drain coastal households’ capital reserves by necessitating high-cost repairs for damaged dwellings and assets (Atiglo and Codjoe, 2017), disrupting fishing activities (Rahman and Schmidlin, 2014), or inducing health issues which lower household productivity (Burney and Naylor, 2012; Rao et al., 2020). Climate hazards can also impact SWB; for example, flooding can induce anxiety (Sekulova and Van den Bergh, 2016), and increase relative comparability when unequal exposure and adaptive capacity creates “winners and losers” (Adger et al., 2020). Additionally, it is increasingly recognized how “happiness” can foster a “resiliency culture” (Addai et al., 2014, p. 874) amidst the growing economic, social and environmental challenges experienced in LMICs. Therefore, lower community SWB and cohesion could, in turn, feedback to further increase environmental vulnerability (O'Brien, 2013).

Landscape characteristics can also affect OWB and SWB. For example, remoteness from urban hubs could curtail OWB by restricting market access (Ackah, 2013), whilst also impacting SWB by controlling individuals’ comparative reference points (Ballas et al., 2007; Ravallion, 2014). Different landcover properties may also influence wellbeing by providing communities with ecosystem services, such as freshwater sources improving sanitation (Pickering and Davis, 2012; Ngure et al., 2014), and by governing social norms/practices (Fagerholm et al., 2016). For example, collectivism and togetherness are often associated with areas abundant in ecosystem services, such as agricultural land (Schaafsma and Gross-Camp, 2021).

Wellbeing is also recognized as a “means” to further wellbeing, with OWB and SWB capable of influencing “adaptive capacity” (Weis et al., 2016). For example, education (OWB) facilitating the implementation of irrigation to mitigate drought impacts (Muttarak and Lutz, 2014), and place attachment (SWB) affecting decisions regarding seasonal or long-term migration (Adger et al., 2013; Amoako et al., 2021).

Despite focusing on environmental conditions, “environment” is one of many contextual components, alongside social, political and economic pressures, which may influence individuals’ wellbeing (Scott, 2006).

## 3 Materials and methods

### 3.1 Study area

**Figure 2:**
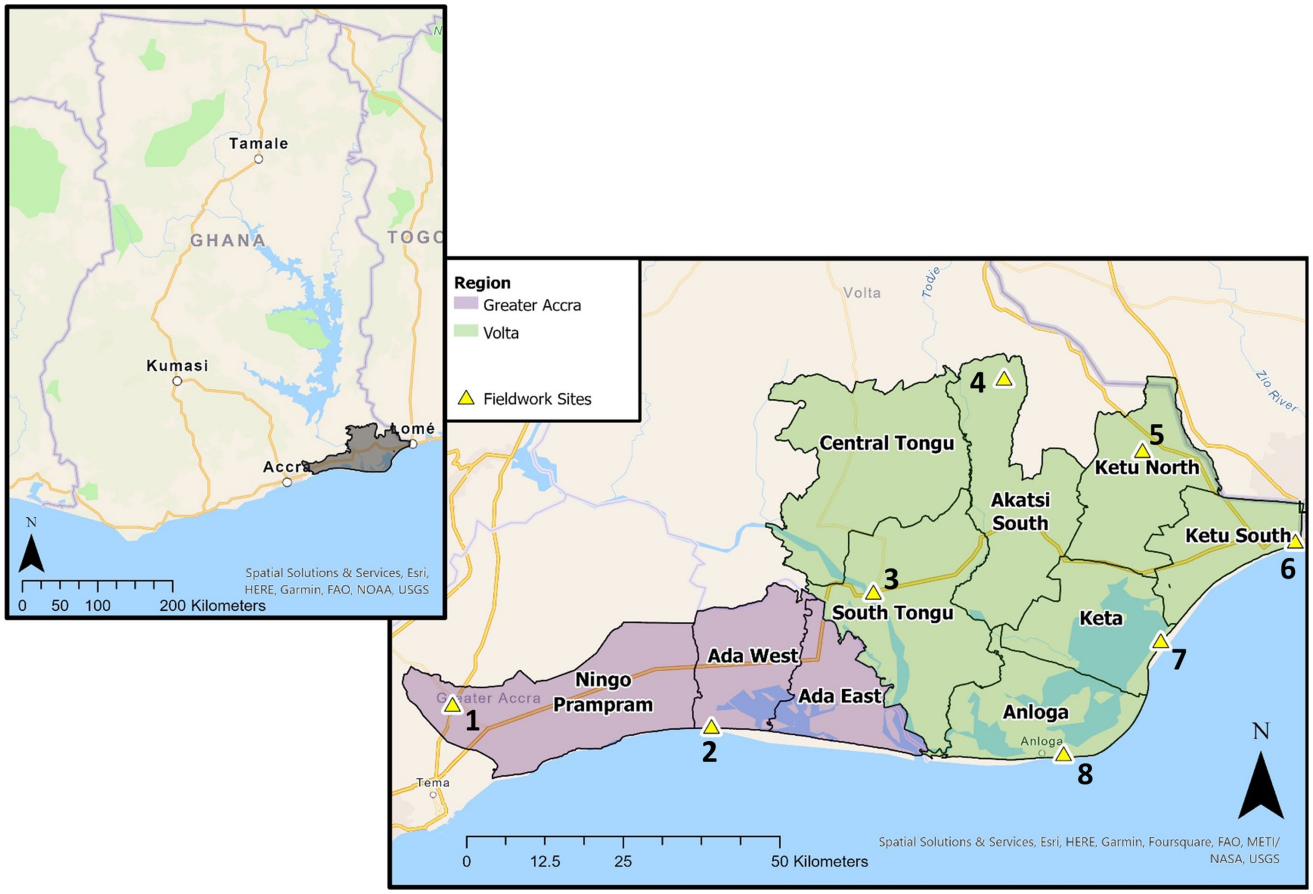
Map of Volta Delta, with regional and district boundaries. Eight selected communities where PRA methods were undertaken, situated within eight different districts, are also illustrated. The offset GPS coordinates illustrate Nyitawuta (Site 4) to be within the Akatsi South district; however, upon arrival we were informed it falls under the jurisdiction of the Akatsi North district.

Volta Delta is located across two regions, Volta and Greater Accra (Figure 2). Landcover predominantly consists of cropland, grassland, wetland and lagoons, with a 27 × 2 km spit separating Keta lagoon from the ocean. Volta Delta contains 4% of the national population (945,827), with population density greater than the national average (151 v 103 people/km^2^). However, population growth is lower than the national average (1.6% vs. 2.1%/yr. [2000–2010]), driven by limited land access, increasing degradation and dwindling fishing opportunities causing high emigration (−41,000 net migration [2000–2010]). However, migration is spatially variable, with positive net migration in Ada West, Ada East and South Tongu districts due to diverse opportunities within tourism, commercial oil/salt extraction and aquaculture (Addo et al., 2018b).

Households are primarily Christian, with Traditional religions and Islam also practiced across the two major ethnic groups; Ewe and Ga-Dangme. Both groups are patrilineal, meaning assets and land rights are inherited along male bloodlines, resulting in gendered imbalances in financial, physical and human capital (Kutsoati and Morck, 2014; Codjoe et al., 2020). For example, educational attainment in Volta Delta is low, with 30% 15+ year olds being illiterate, yet literacy levels are higher amongst males (GSS, 2013).

Primary sector livelihoods, involving natural resource extraction, contribute most to delta GDP (29%); agriculture (22%) and fishing (7%). Trade, transport and industry (inc. salt mining and food processing) contribute 20% GDP each, and construction 11%. Approximately 1/3 individuals work within agriculture, higher than the proportion of GDP generated. This disparity is driven by high subsistence farming, low productivity and limited technology access curtailing monetary returns (Arto et al., 2020).

As discussed in section (2.3), environmental hazards can impact wellbeing through various mechanisms, particularly within the primary sector. The prominence of subsistence agriculture accentuates the sensitivity of livelihoods and wellbeing to environmental challenges. Volta Delta is exposed to multiple hazards including; warming temperatures, drought (Niang et al., 2014), uncertain monsoon timings, increased rainfall intensity, riverine/coastal flooding, and storm surges (Codjoe et al., 2020). Furthermore, the Accra coast is experiencing sea-level rise, estimated at 3.3 mm/yr (1974–2005) (Sagoe-Addy and Addo, 2013), exacerbated by land subsidence (1–2 mm/yr.) (Syvitski, 2008) following extensive groundwater extraction (Addo et al., 2018a). Frequent flooding also contributes towards coastal erosion, estimated at 0.53 m/yr (1986–2013) (Addo, 2015).

Volta Delta was selected for this study due to the multitude of environmental challenges and landscapes providing a space to research how different mechanisms may influence varying elements of wellbeing. Furthermore, despite national financial poverty being concentrated within North Ghana (World Bank, 2020), southern Volta is one of five regions with an increasing (2012–16) extreme poverty rate (11.4%) higher than the national average (8.2%) (GSS, 2018). The divergence from the national trend provides an opportunity to explore potential drivers of low OWB within a vulnerable coastal environment. Additionally, Volta and Greater Accra regions possess different histories, industries, landscapes, and investment levels (Awanyo and Attua, 2018), facilitating the exploration of how SWB spatially varies across the OWB spectrum. For example, Greater Accra had a 0.0% extreme poverty rate in 2016/17. This regional disparity is linked to (post) colonial investment patterns, with Greater Accra favored due to its rich supply of natural resources and the importance of the port in the global cocoa trade (Kambala, 2022).

### 3.2 Data

This section summarizes the survey and remote sensing datasets used within binary logistic modelling. Details on qualitative data collection are presented in section (3.4.2).

#### 3.2.1 Survey dataset

Data was collected through the Deltas, Vulnerability and Climate Change: Migration and Adaptation (DECCMA, 2020) survey. The survey was undertaken with household heads using face-to-face interviews (April–June 2016). A two-stage clustered sampling strategy aimed to survey 1,500 households. Households were stratified into five strata based on environmental risk. Fifty enumeration areas, classified during the 2010 Census, were randomly selected, proportional-to-size, from the strata^2^. Thirty occupied residential dwellings from each enumeration area were randomly selected. A 91% response rate was achieved (1,364 households), ensuring the sample is representative of the Volta Delta population. Further information on the survey strategy is available in Atiglo et al. (2022), where the secondary data source was used to investigate coastal vulnerability.

The survey collected various data, including households’ finances, subjective evaluations, self-reported climate hazards, and sociodemographic characteristics. The self-reported hazard variables include; shocks (floods and storms) and stresses (drought, salinization and erosion). Respondents recorded their (i) “exposure”; the hazard is experienced on an annual, or more-frequent, basis (ii) “environmental impact”; negative impact upon at least one of housing, health, water or food (iii) “economic impact”; negative impact upon economic security and/or crops and livestock. See Supplementary material S1 for further information on variable coding.

#### 3.2.2 Remote sensing

GPS points for each enumeration area facilitated the spatial join between survey and remote sensing data. Multiple remote sensing datasets were collected to create contextual environmental variables.

Firstly, landcover information was created from LANDSAT-7 30 m resolution images, using FAO Land Cover Classifications (Jayson-Quashigah, 2016). The proportion of each landcover classification was calculated for each community buffer^3^. Existing health studies in Ghana using 2 km buffers (i.e., Krefis et al., 2011), the urban/rural mix within Volta Delta (Perez-Heydrich et al., 2013), and the preference for minimal overlap between communities’ associated environments, justified the application of 2 km buffers throughout. Nevertheless, the limitation of individuals accessing environments outside their immediate community is acknowledged (Douxchamps et al., 2015).

Further landscape variables were collected from WorldPop (2016) Geospatial data, including; distance from major roads, road intersects (Christiaensen et al., 2003) and inland water^4^. “Remoteness” variables were collected using Google API data, including travel time^5^ between each enumeration area centroid and both Accra and district capitals. Travel time, rather than Euclidean distance, is more effective in capturing remoteness in locations where accessibility is restricted by coastlines or physical barriers (i.e., rivers) (Boscoe et al., 2012).

### 3.3 Model variables

#### 3.3.1 Independent variables

Multiple independent variables, incorporated within regression models, were constructed from survey and remote sensing datasets. This paper focuses on environmental variables, both self-reported climatic hazards and landscape characteristics, whilst also recognizing the influence of control variables on wellbeing outcomes. See Supplementary material S1 for information on all “environmental” and “control” variables.

#### 3.3.2 Dependent variables

Wellbeing is measured in numerous ways across disciplines and contexts (White, 2016). To address the research aim, this study calculates expenditure poverty (OWB) and life domains unhappiness (SWB) to reflect opposite ends of the objective-subjective spectrum.

##### 3.3.2.1 OWB (expenditure poverty)

OWB was measured using a neoclassical, financial approach. This approach was selected due to the greater data availability and simpler interpretation. Additionally, it aligns with the key targets for SDG1, and represents the most common, arguably most universally recognized, objective measure (Laderchi et al., 2003).

Financial OWB was calculated using expenditure rather than income (Atkinson, 2019). Expenditure is defined as the value of “goods and services [used] to directly satisfy a person’s [immediate] needs” (OECD, 2013, p. 41). Financial OWB is commonly defined by income in HICs where most work in formal employment with up-to-date financial records, contrasting LMICs with informal employment, poor records and non-monetary payments. Additionally, LMIC incomes are volatile due to seasonal labor and climatic uncertainty (Atkinson, 2019). Therefore, income measures within LMICs may produce inconsistent results. For example, households may experience poverty during fallow periods, yet not during harvests. Furthermore, income is vulnerable to survey error due to recall and social desirability bias, and difficulties in quantifying non-monetary payments (Haughton and Khandker, 2009). In contrast, expenditure can produce less-erratic results as it incorporates households’ use of savings and loans to maintain living standards (Davis, 2014), whilst World Bank (2005) also suggests survey errors are lower for expenditure estimates in LMICs.

Each household’s recorded expenditure types were summed, excluding house rent and loan repayments (Supplementary material S2.1). Renting costs were removed to avoid incorrectly identifying homeowners as experiencing poverty, and loan repayments were excluded as they insufficiently relate to living standards (Arndt and Tarp, 2016). Household expenditure was adjusted for regional living costs (Supplementary material S2.2), and equivalized using the Ghanaian Statistical Service (GSS, 2018) scale (Supplementary material S2.3) to provide “expenditure/equivalent adult per year.” Equivalisation facilitated comparison between households (Batana and Cockburn, 2018) by accounting for differences in household size, age composition and “economies-of-scale” (OECD, 2011).

Adjusted, equivalized household expenditure was compared to the GSS upper poverty line. The threshold (GH¢1,760.8 adult equivalent/year) is based on costings of essential food and non-food items, such as cleaning products and energy. Households with expenditure below the threshold were classified as experiencing poverty.

##### 3.3.2.2 SWB (life domains unhappiness)

“Happiness” and “life satisfaction” are commonly used interchangeably when defining SWB; however, Kahneman and Deaton (2010) note a distinction between everyday happiness, driven by emotions, and longer-term satisfaction, incorporating thoughts around one’s life. Nevertheless, Cooke et al. (2016) claims the terms are indistinguishable, and using a single term minimizes confusion. Therefore, this study refers to “happiness” throughout.

Happiness was captured using a life domains approach, which assumes happiness with different components additively represents overall happiness (Rojas, 2006). This approach was selected over a “global” approach, which captures “general” happiness, as studies suggest life domains responses draw on different information and specific experiences, compared to “abstract” global evaluations (Schwarz and Strack, 1999; Kozma et al., 2000; Cummins et al., 2003; Lent, 2004). Therefore, “life domains” approaches are arguably more capable of capturing less-tangible wellbeing components, such as community satisfaction (Fagerholm et al., 2016). For example, 78% generally unhappy households in the DECCMA survey were happy with “community interactions,” yet only 11% were happy with “more-tangible” economic security (Supplementary material S3).

Ordinal principal component analysis was used to agglomerate 8 correlating Likert scale measures of happiness; incorporating household heads’ happiness with financial, material, social and environmental components. The first component was used as the happiness index (Berni et al., 2011), with all domains positively correlated to the index (Supplementary material S4). Next, k-means clustering was used to formulate low, medium and high happiness clusters (Vyas and Kumaranayake, 2006). The “low” cluster was defined as “unhappy” in the binary outcome variable. Transforming the index into a binary variable enabled comparability with expenditure poverty and reduced the significance of small index differences which may have resulted from measurement error (Lutz, 2022). Despite studies suggesting individuals can comprehend “happiness” and “unhappiness” differently (Uchida and Kitayama, 2009; Kobayashi and Hommerich, 2017), this study interprets them as two ends of a single continuum; aligning with the DECCMA survey which presented “very unhappy” and “very happy” on the same Likert scale.

The variable was constrained by the available data, with SWB information often limited within survey datasets (Sulemana et al., 2016). This limitation justified the inclusion of qualitative methods to understand the complex processes behind static quantifiable results (Shaffer, 2013), gain additional insights regarding local values and interpretations (Doyle et al., 2016), and allow for other conceptualizations, such as “eudaimonic SWB,” to be explored. Furthermore, to facilitate analytical comparability, this study assumes household heads’ SWB represents the whole household. Therefore, qualitative analysis is also pivotal in understanding individuals’ SWB and intrahousehold differences.

##### 3.3.2.3 Combined OWB/SWB outcomes

To understand the relationship between OWB and SWB, the two measures were combined to create four additional binary outcomes (Table 1). This paper focuses on “opposing” “poor/happy” and “non-poor/unhappy” outcomes^6^.

**Table 1:** Two outcome variables incorporated in regression models (highlighted gray).

		Life domains unhappiness (SWB)	
		No	Yes
Expenditure poverty (OWB)	No	“Non-poor and happy”	“Non-poor and unhappy”
	Yes	“Poor and happy”	“Poor and unhappy”

Constructed using binary objective expenditure poverty and subjective life domains unhappiness measures.

The spatial distributions of “poor/happy” and “non-poor/unhappy” households are also illustrated to demonstrate how wellbeing varies across Volta Delta. The proportion of households experiencing the two outcomes, within all 50 sampled enumeration areas, was calculated and compared to the sample average.

### 3.4 Methodology

The study followed an explanatory sequential mixed methods approach, where quantitative results guided fieldwork site selection and broad question design, with qualitative results used to validate or challenge statistical outputs (Bryman, 2006). Incorporating qualitative methods was crucial in understanding SWB, as interacting with local communities restores the “person” into analysis, which could have otherwise been lost if exploring SWB solely via external, quantifiable surveys (White, 2016). Mixed method approaches can also enrich findings by aiding the interpretation of counter-intuitive quantitative results and the direction of causality (Place et al., 2007; Shaffer, 2013; Rao et al., 2017). Despite quantitative methods primarily guiding the study, integrating qualitative elements throughout the research process prevents results from being interpreted as tokenistic add-ons (Doyle et al., 2016).

#### 3.4.1 Quantitative modeling

Two binary logistic regression models were constructed using DECCMA survey data to explore how environmental conditions associate with “poor/happy” and “non-poor/unhappy” outcomes. Multiple control characteristics were also incorporated to account for non-environmental effects. From a methodological perspective, the use of binary rather than multinomial modelling improved model parsimony and interpretability. This approach allowed for specific OWB/SWB combinations to be compared to the rest of the sample, rather than an arbitrary reference group (Mahmood et al., 2019).

Single-level models, using forward stepwise selection, were selected due to between-cluster variation being accounted for by inputted variables (Groves et al., 2011). Variables were inputted in order based on variable significance and AIC within preliminary bivariate models; significant environmental, significant control, non-significant environmental, and non-significant control. Model assumptions were satisfied, including multicollinearity (Pan and Jackson, 2008) and linearity between numerical variables and log odds of dependent variables.

Interaction terms, focusing on environment-control relationships, were also tested to capture information regarding “adaptive capacity” (Reisinger et al., 2020). However, no interaction terms were significant in either model. Results are presented as odds ratios (exp[β_k_]), whilst controlling for all other variables in the model. Interpreted results are significant at the 5% level.

#### 3.4.2 Qualitative methods

Participatory rural appraisal (PRA) methods, including semi-structured focus groups (FGs) and interviews (Schreckenberg et al., 2016), were undertaken across eight communities (Figure 2). Discussions were centered around local understandings of “wellbeing,” actors’ experiences of climate hazards, and how environmental conditions influenced their ability to fulfil their basic needs and wellbeing priorities.

“Wellbeing” is commonly interpreted as “good health” or “good character” in Ghana (Osei-Tutu et al., 2020); therefore, following discussions with local research assistants (RAs), and engaging with the literature (Dzokoto et al., 2019), respondents were alternatively asked about what was required for “a good life.” This terminology ensured respondents incorporated broader concepts of wellbeing, with previous Ghanaian studies illustrating a “good life” to include less-tangible elements such as peace of mind, working in unity and fulfilling work-based identities (Osei-Tutu et al., 2020). Defining wellbeing through a “good life” ensured the interpretation was relational, and not imposed by external assumptions (Gough and McGregor, 2007).

Communities were selected based on key themes from quantitative modelling, including areas with distinct wellbeing outcomes, landscapes and climatic characteristics. For example, Awlikope (Site 5) was selected due to high cropland coverage, Nyitawuta (Site 4) due to a high proportion of households recording low OWB/high SWB, and Kedzi (Site 7) due to flooding and erosion exposure.

Despite visiting the same communities, the survey and fieldwork respondents were different. Furthermore, it is recognized that qualitative fieldwork was conducted 7 years after DECCMA. Therefore, due to contextual changes, such as the national economic downturn (World Bank, 2022; IMF, 2023) and global pandemic, qualitative findings are used to supplement the discussion, rather than explain quantitative results. Based on the explanatory sequential approach, qualitative results, primarily in the form of verbatim quotes, will be presented in the “Discussion” (section 5) to aid the interpretation of model results and enrich quantitative findings with locally grounded perceptions.

##### 3.4.2.1 Focus groups

Two FGs were carried out at each site; one female and one male (Table 2). Female and male RAs led the corresponding FGs to ensure participants were comfortable disclosing information (Yager et al., 2013). Furthermore, unlike household-level quantitative analysis, gendered FGs enabled intrahousehold differences in wellbeing and environmental experiences to be explored. However, power dynamics along other social lines, such as political influence or age, could still have generated bias (Farr, 2018). See Supplementary material S5 for additional FG information, including participants’ age profiles. Despite discussions with local gatekeepers regarding the importance of selecting participants with representative characteristics, certain voices (i.e., lower educational levels) may have been seldom heard in some locations (Tsekleves et al., 2020).

**Table 2:** Number of focus group and interview respondents, including gender, across the eight selected communities.

	Focus groups		Interviews	
Location	No. male participants	No. female participants	No. community participants	No. DPO participants
1. Afienya	7	6	1 M	1F
2. Anyamam	8	8	1M, 1F	1F
3. Sogakope	6	10	1M, 1F	1 M
4. Nyitawuta	8	8	1M, 1F	1 M
5. Awlikope	8	8	1M, 1F	1 M
6. Aflao	7	8	1M, 1F	1 M
7. Kedzi	9	7	2M	1 M
8. Anloga	10	8	1M, 1F	1 M

Due to most participants being unable to read English, RAs translated consent forms to allow respondents to provide written consent. All FGs were undertaken in Ewe or Dangbe, except the male FG in Afienya (Site 1) where discussions were in English.

Semi-structured FGs, which incorporate pre-determined topics, yet allow “flexibility for participants to bring their own personality and perspective to the discussion” (Barrett and Twycross, 2018, p. 63), allowed collective understandings of wellbeing to be explored, and ensured respondents’ own conceptualizations were prioritized (Wilkinson, 1998). See Supplementary material S6 for site-specific FG question guides.

##### 3.4.2.2 Semi-structured interviews

Two semi-structured interviews were also conducted at each site^7^ with individuals encountered during FGs or community walks. Individuals were selected based on the interest in their FG inputs and their livelihood, particularly if similar livelihoods were not selected for FGs. Semi-structured interviews, loosely centered around environment-wellbeing themes uncovered during regression analysis, ensured participants could freely describe their environmental experiences, rather than being restricted by researchers’ assumptions.

Interviews were carried out following FGs to access more-detailed information on environment-wellbeing associations (Guest et al., 2017), particularly regarding SWB which is more accessible, and easier to probe, in one-to-one settings (Kvale and Brinkmann, 2009). Interviews also provided the opportunity to explore individuals’ comparative reference points, which were hypothesized to strongly influence SWB (Ravallion, 2014). Undertaking interviews after FGs also provided an opportunity to detect differences in individualistic/collective wellbeing conceptualizations, and to evaluate whether social desirability impacted responses in group settings (Groves et al., 2011). See Supplementary material S6 for the general interview guide, and female-specific questions. Additional *ad-hoc* questions were also posed to respondents based on previous FG responses and community walk observations.

Eight District Planning Officers (DPOs) were also interviewed face-to-face or via telephone^8^. These interviews were flexible, drawing on key themes from community FGs. As DPOs coordinate and monitor development plans across multiple governmental institutions, such as health, education and employment, the interviews enabled broader discussions regarding district-level challenges and policy decisions.

All FG and interview discussions were audio recorded and transcribed. Following transcription, thematic analysis was undertaken, with codes created inductively and deductively (Fereday and Muir-Cochrane, 2006). The aim was not to quantify codes, but to extract key themes and verbatim quotes across the study sites to support or challenge quantitative results.

## 4 Results

### 4.1 Wellbeing rates

Due to the measures originating from different disciplines, and containing different information, varying rates were produced. 506 households (37%) experienced objective expenditure poverty, whereas 202 households (15%) were subjectively unhappy.

Next, examining combined measures, most households (*n* = 744, 55%) were “non-poor/happy.” Yet, focusing on opposing outcomes, 110 households (8%) were defined as “non-poor/unhappy,” and 415 households (30%) as “poor/happy.”

### 4.2 Spatial distribution

**Figure 3:**
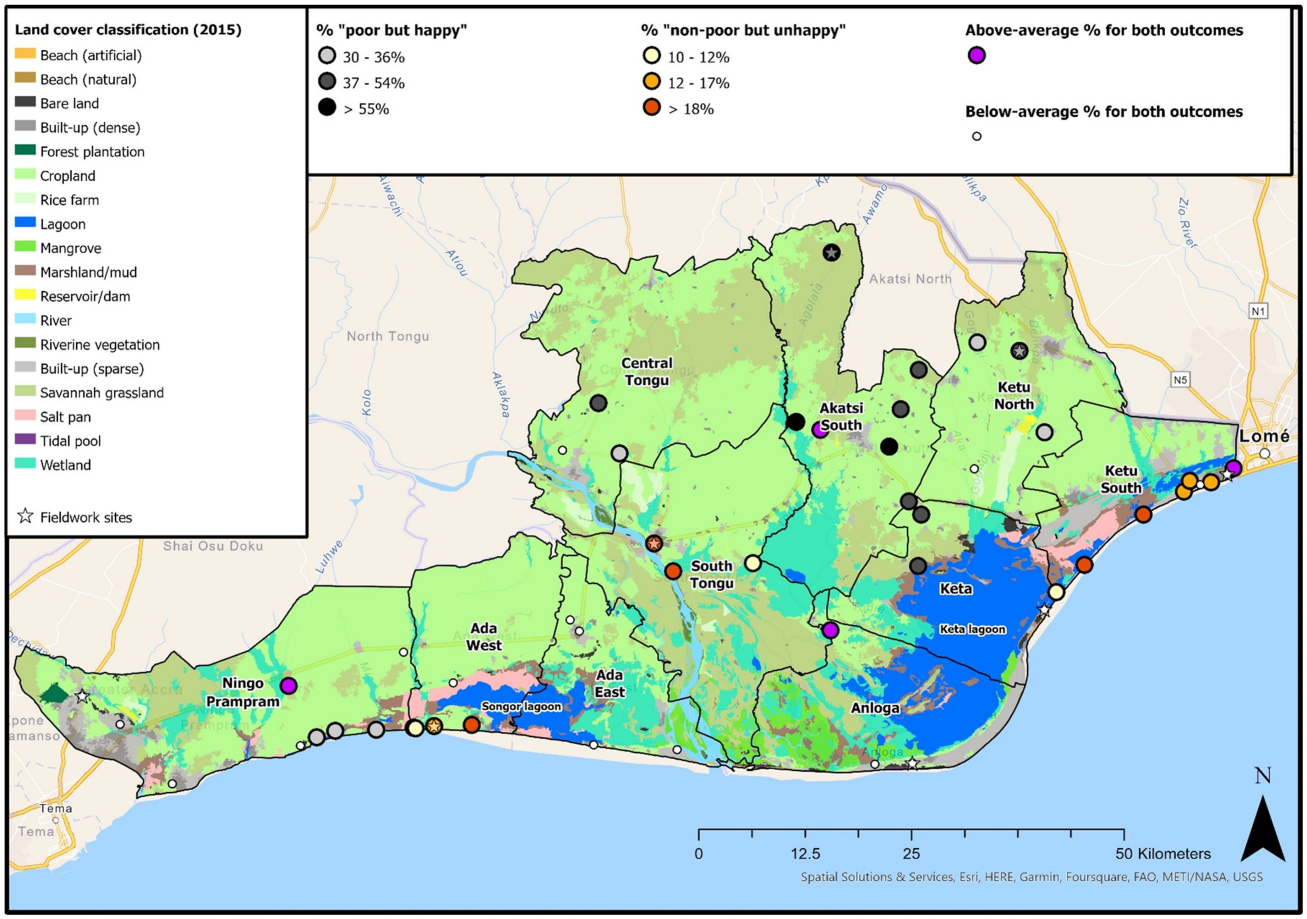
Proportion of households in each surveyed enumeration area classified as “non-poor/unhappy” (red) and “poor/happy” (gray). Enumeration areas with “above-average” proportions are highlighted. Enumeration areas with “above-average” proportions for both outcomes are highlighted purple, and highlighted white if the proportion is “below-average” for both outcomes. Landcover data (Jayson-Quashigah, 2016) and study sites also presented (see Table 2 for site names).

“Non-poor/unhappy” households are concentrated in three areas; close to Sogakope (Site 3), near the Togo capital (Lomé), and south of Songor Lagoon in Ada West; the latter including beach and salt pan coverage. In contrast, “poor/happy” households are concentrated in inland, agricultural/grassland communities in Akatsi South and Keta (Figure 3).

### 4.3 Model results

Descriptive statistics of the explanatory variables included in the final regression models are presented in Supplementary material S7 to provide a profile of the DECCMA sample.

Model 1 showed environmental conditions to significantly account for variation in the “poor/happy” outcome (Table 3). “Low,” “medium” and “high” cropland coverage within communities increased odds 229, 149 and 199% compared to those with no cropland, whereas the presence of riverine vegetation, primarily located around Sogakope (Site 3), reduced odds 66%. Climate hazards also associated with the outcome, with households economically impacted by drought and environmentally impacted by erosion having odds 49 and 54% higher respectively, whereas economic flood impacts reduced odds 44%. Higher odds were also recorded for larger households, with open-source drinking water, non-flushing latrines, current migrants and unschooled household heads.

**Table 3:** Model 1 – binary logistic regression model results; “poor/happy” outcome.

**Variable**	**Odds coefficient [exp(β)] (S.E)**
**Climatic shocks and stresses**	
Economic impact from drought (ref: No impact)	
Impacted	1.493 (0.261)**
Economic impact from flooding (ref: No impact)	
Impacted	0.563 (0.116)***
Environmental impact from erosion (ref: No impact)	
Impacted	1.540 (0.271)**
**Environmental landscape/remoteness**	
Region (ref: Greater Accra)	
Volta	3.628 (0.792)***
Cropland in 2 km community buffer (ref: None)	
Low coverage	3.293 (0.866)***
Medium coverage	2.488 (0.742)***
High coverage	2.956 (0.724)***
River vegetation in 2 km community buffer (ref: No)	
Yes	0.339 (0.113)***
**Physical assets**	
Drinking water source (ref: Piped or tubewell)	
Dug well or open-source	1.841 (0.326)***
Latrine facility (ref: Flushing)	
Pit/Public/KVIP	2.107 (0.797)**
No facility	3.319 (1.296)***
**Household characteristics**	
Household size (ref: 1-person household)	
2–3 people	4.072 (1.268)***
4–5 people	11.053 (3.398)***
6–7 people	14.995 (4.926)***
8+ people	32.438 (12.156)***
**Household head characteristics**	
Highest education of household head (ref: No schooling)	
Primary education (below-basic)	0.775 (0.143)
Lower secondary education (basic)	0.854 (0.166)
Higher secondary or higher education (above-basic)	0.376 (0.094)***
**Adaptations**	
Current migrant out of household (ref: No)	
Yes	1.471 (0.220)***
**Additional model information**	
Intercept	0.003 (0.002)***
No. observations	1,237
Log Likelihood	−600.313

^***^p < 0.01, ^**^p < 0.05, ^*^p < 0.1 (significance level).

Model 2 examined significant associations with “non-poor/unhappy” households (Table 4). Three landscape characteristics associated with the outcome. “Low” and “high” cropland coverage reduced odds 55% compared to areas with no cropland, whereas the presence of riverine vegetation and wetland landcover increased odds 286 and 121%, respectively. Furthermore, climate hazards, including environmental salinity impacts and flood exposure, increased odds 117 and 100%, respectively. Lastly, higher odds were recorded for smaller households with lower place/community attachment (see Supplementary material S8 for methodology).

**Table 4:** Model 2 – binary logistic regression model results; “non-poor/unhappy” outcome.

**Variable**	**Odds coefficient [exp(β)] (S.E)**
**Climatic shocks & stresses**	
Environmental impact from salinity (ref: No impact)	
Impacted	2.167 (0.531)***
Exposed to flooding (ref: Not exposed)	
Exposed	1.999 (0.461)***
**Environmental landscape/remoteness**	
Cropland in 2 km community buffer (ref: None)	
Low coverage	0.454 (0.139)***
Medium coverage	0.866 (0.274)
High coverage	0.446 (0.163)**
River vegetation in 2 km community buffer (ref: No)	
Yes	3.861 (1.436)***
Wetland in 2 km community buffer (ref: No)	
Yes	2.210 (0.816)**
**Subjective characteristics**	
Place/community attachment (ref: Low attachment)	
Medium attachment	0.772 (0.220)
High attachment	0.496 (0.144)**
**Household characteristics**	
Household size (ref: 1–4 person household)	
5+ people	0.528 (0.132)***
**Additional model information**	
Intercept	0.067 (0.033)***
No. observations	1,275
Log likelihood	−313.102

^***^p < 0.01, ^**^p < 0.05, ^*^p < 0.1 (significance level).

## 5 Discussion

Despite most households experiencing aligning “non-poor/happy” outcomes, it remains important to investigate opposing outcomes within specific locations and livelihood groups to avoid results masking local challenges or being generalized across the study area. Furthermore, wellbeing, and its association with environmental conditions, is relational to a specific time and place (Gough and McGregor, 2007). Therefore, the prominence of aligning OWB/SWB may not necessarily be a permanent feature, especially when acknowledging the different objective and subjective challenges arising from increasing environmental pressures. Consequently, understanding why OWB/SWB may oppose one another can help uncover the potential interventions required to ensure communities experience comprehensive wellbeing under current, and future, conditions.

Quantitative models, and the spatial distribution of wellbeing (Figure 3), unveil a broad distinction between the environmental conditions associated with “non-poor/unhappy” and “poor/happy” outcomes. “Non-poor/unhappy” households are primarily in non-agricultural areas, including built-up riverine towns and coastal areas exposed to salinity/flooding. In contrast, “poor/happy” households are primarily situated within inland, agricultural locations impacted by drought/erosion.

This section discusses the significant environmental associations recorded in Models 1 and 2. These results are complimented and/or challenged by community and DPO responses, drawing on the most prominent themes developed during thematic analysis:

- Climatic risk (damage and fears)
- Remoteness and accessibility
- Social norms, security and status
- Place attachment
- Social capital (collective/individualistic)
- Aspirations and relative/past comparisons

As mentioned, the study did not aim to quantify qualitative codes, but rather use broad themes and verbatim quotes^9^, to further explore quantitative outputs. Therefore, this discussion will incorporate dialogue from interviews and FGs, alongside evidence from the literature, to interpret the regression model results.

### 5.1 “Poor but happy” households

This section explores potential mechanisms behind low OWB/high SWB in vulnerable agricultural locations. Discussions are structured around Model 1 results, focusing on cropland landscapes, drought/erosion impacts and physical remoteness.

#### 5.1.1 Landscape: cropland coverage

The association with cropland communities is based upon statistical results (Model 1), and not a simple perpetuation of the “poor but happy” stereotype commonly portrayed in LMIC research and mass media (Kay and Jost, 2003). Happiness should not be considered a substitute for financial capital; people do not “love squalor, they make do with what they have” (Dowling and Yap, 2012, p. 4). Therefore, this section highlights the benefit of incorporating OWB *and* SWB within development policy.

Opposing low OWB/high SWB may have been generated by agricultural communities placing less emphasis on financial OWB when self-evaluating their happiness. For example, communities may strive for the financial security required to fulfil non-material needs such as community relations and cultural identity, rather than accumulate capital for increased market activity (Ward and King, 2016).

“in rural areas, money is not their goal, their goal is having the social network … the communal feeling of working [whereas urban dwellers] everything to get income … when you have resources … people adore you” (3,DPO)

“Even though I am not rich I am happy, I do not want to overburden myself thinking about my lack of money” (4,FG,M)

Fulfilling the intergenerational “crop farmer” identity within these landscapes, and providing for the family and community (Markussen et al., 2018), was also suggested to produce happiness by bringing a sense of pride (Brown et al., 2021), regardless of objective financial security. The importance of preserving traditional family livelihoods was exemplified by a young man in Nyitawuta (Site 4), who claimed they would return to the rural village to undertake their farming duties, even if they fulfilled their aspirations of attending university. This influence upon SWB is particularly pertinent amongst men, with previous studies showing the respect received from fulfilling “masculine breadwinner” responsibilities (Carr, 2008).

“The inability to take care of your wife and children causes heartache to the man of the house” (5,FG,M)

The importance of working within society, despite continued climatic hazards, was further illustrated by multiple respondents claiming unemployed or “lazy” community members had rejected social norms (Grischow, 2008), with “discrepancies between reality and culturally-sanctioned values [a] source of societal concern” and lower SWB in Ghana (Dzokoto, 2012, p. 319).

“We are farmers that is our identity … Sometimes the farming occupation does not go as planned but we never give up” (4,FG,M)

High place attachment, “bonding” social capital (Claridge, 2018), and “relatedness” (Deci and Ryan, 2012) are also crucial conduits for SWB in rural Ghana (Ayerakwa et al., 2015). “Relatedness” is particularly pronounced within spaces of ecosystem services, such as cropland, which provide the platform for social interaction (Fagerholm et al., 2016) and knowledge-sharing (Schaafsma and Gross-Camp, 2021). An attachment to one’s land, commonly passed down generations in rural Ghana (Addo et al., 2018b), can also bring security, peace of mind (Osei-Tutu et al., 2020), and happiness to communities (Gross-Camp et al., 2019).

“I am very proud to be associated with … the land of my youth, I have lived here and have my possessions here and it is just natural … it becomes some sort of pride in you” (5,FG,M)

#### 5.1.2 Climate hazards: drought and erosion

This discussion uses the term “hazard,” rather than “risk,” as the IPCC (2024) defines the latter as having the “potential for adverse consequences for human or ecological systems.” However, by exploring possible mechanisms behind opposing OWB/SWB, this paper acknowledges how different hazards may have negative, positive or neutral influences on varying wellbeing outcomes.

Despite the potential for improved SWB within cropland communities, higher odds of being “poor/happy” (Model 1) when impacted by drought and/or soil erosion suggest there are also multiple mechanisms in which hazards can induce objective poverty within agricultural landscapes: (i) lowering soil moisture and nutrient retention (Garrity et al., 2010) (ii) shortening farming seasons (Antwi-Agyei et al., 2018) (iii) reducing irrigation and hydropower resources (Kaygusuz, 2011) (iv) health issues, such as malnutrition, and illnesses related to poor water quality (Ngure et al., 2014; FAO, 2019) (v) livestock mortality (Serdeczny et al., 2017). Communities also mentioned how shifting rainfall patterns can devalue traditional farmer knowledge (Jennings and Magrath, 2009; Sarku et al., 2020), increase food import costs (Hertel et al., 2010), and restrict the capacity to accumulate capital for productive and/or adaptive investments (Davis, 2014).

“drought has badly affected me … last year I cultivated two acres of cassava and it rained generously so I had … about 15–20 sacks … with this I invest 2000 cedi. But I cultivated the same acres this year and got about 3 bags … the money invested is totally lost” (4,CI,M)

However, despite inevitable financial impacts from drought and erosion, persistent hazards can create “acceptance,” where negative impacts upon SWB are lessened compared to communities without prior experience (Baruah and Hussain, 2021).

“drought has become part of their lives, whether it rains or not, they stay in the same position” (5,CI,M)

Respondents also noted how religion was used to increase their environmental resiliency and maintain SWB. For example, fluctuating rainfall patterns were accepted as “God’s will,” therefore individuals’ worries regarding how to address climatic challenges were mitigated. However, accepting hazards as part of “God’s plan” could discourage tangible adaptive or productive investments; potentially contributing towards “poor/happy” outcomes (Ndamani and Watanabe, 2015; Evadzi et al., 2018; Assan et al., 2020).

“The climatic happenings will always be with us, it is God who created it like that so we will just have to thrive through it to achieve what we want” (3,FG,M)

The OWB impacts from drought and erosion may also stimulate social cohesion as a coping mechanism (Dussaillant and Guzmán, 2014), through an “in it together” attitude (Jordan, 2015; Calo-Blanco et al., 2017; Jensen and Tiwari, 2021). Frustrations with surrounding vulnerability or injustice may trigger collective action (Cattell, 2001), enhancing SWB by providing channels for emotional support (Lent, 2004).

“if someone is affected by rain, erosion, drought, the community … assists the person, people … bring up ideas that will be of great help … we do this because of unity, love, peace for each other” (4,FG,M)

However, community-wide support within agricultural locations could reinforce low OWB/high SWB, as persistent sharing could limit the surplus capital available for individuals to invest and escape poverty (Di Falco and Bulte, 2009).

“[hazards] become a burden on us all because the affected person borrows money to survive” (8,FG,M)

Discussions with select communities did, however, suggest model results were not generalizable. For example, persistent social capital within agricultural communities, often associated with stereotypical “poor but happy” identities (Kay and Jost, 2003), was shown to potentially be romanticized, as community togetherness fluctuated within the vulnerable environmental context (Adler, 2015; Craig et al., 2022). Select respondents noted how financial insecurity during droughts could weaken community bonds, with material support withheld to ensure resources were available to overcome their own challenges.

“people who were not affected will hesitate in supporting the affected people because they do not know when they may also be affected” (5,FG,M)

#### 5.1.3 Landscape: remoteness

The remoteness of agricultural land from road networks and peri-urban hubs (Supplementary material S9), may also contribute towards “poor/happy” outcomes. Firstly, once controlling for cropland coverage, the association between residing within Volta Region and lower OWB (Model 1) could relate to communities having poorer connectivity to profitable markets in Greater Accra Region (i.e., Accra/Tema) (Stifel et al., 2003; Owusu and Lund, 2004; Chamberlin, 2007).

“we look for motorbikes to transport … by the time we get there the customers have left … you will not be able to sell your produce at any substantial price” (4,CI,F)

Moreover, despite national decentralization aiming to reduce rural/urban inequalities, rural locations do not always access similar benefits (Owusu, 2005; Awuah et al., 2009). For example, lower public investment into healthcare within remote areas can feedback to lower household productivity and increase domestic care burdens (Hallegatte et al., 2018); potentially contributing towards expenditure poverty (Model 1).

“there are a lot of inequalities … because this is the district capital, it is expected to have … basic facilities … the capital is developing and ideally it should be duplicating in other communities, but [it is not] because the assembly is [financially] handicapped” (2,DPO)

However, in contrast, remoteness from more-developed locations could contribute towards “poor/happy” outcomes (Model 1) by buffering against negative SWB impacts. SWB is influenced by individuals’ comparisons to various reference points (Ballas et al., 2007). Remote, agricultural locations may be less aware of higher living standards in better-connected urban areas, such as Greater Accra, or perceive them to be less realistically attainable. Therefore, greater collectivism, and lower inequality and relative comparability, could heighten SWB (Corazzini et al., 2011; Ng and Diener, 2014).

“They only compare themselves to others in the community, they cannot compare to others because they are all subsistence farming” (5,DPO)

“if I am privileged to have food at home, I will wish that the same applies to other people in the community … if I am the only happy person and others are in pain it doesn’t feel right” (4,CI,M)

Nevertheless, the assumption of remote communities being unaware of external living standards, or being less impacted by relative differences, was challenged in Nyitawuta (Site 4). Respondents noted how local comparisons can cause “thinking,” which is thought to disturb an individual’s “peace of mind,” a pivotal component of SWB in Ghana; “if you not thinking about anything, you are comfortable” (Osei-Tutu et al., 2020, p. 7).

“**If others are doing better than you, how do you feel?** … I will be sad … Because, why are things going on well with others and it is not going on well with me, so I will be thinking” (4,CI,F)

“I compare myself to the people living in the cities. If I were … in the city I will have a better life … they have good roads, water, jobs … buildings” (4,FG,M)

Even if individuals are aware of higher living standards, accepting that such standards are not realistically attainable, due to remoteness or limited mobility, could mitigate the impact of relative comparability upon SWB. Furthermore, despite recognizing broader inequalities, many were content with their lives and generated SWB through alternate non-market channels. For example, the autonomy and perceived security gained from freely accessing land and resources, and constructing one’s own dwelling, compared to urban areas where payment is required (Danso, 2013).

“the land is free, we have built our small houses and they are for us and not anyone else … We are content because no one is chasing us for rent” …“over here all you have to do is get one or two cups of water to drink, you do not have to pay for it and we are happy” (4,FG,F)

### 5.2 “Non-poor but unhappy” households

This section explores potential mechanisms behind high OWB/low SWB in non-rural, coastal/riverine locations. Discussions are structured around Model 2 results, focusing on non-rural landscapes, remoteness, proximity to water sources, and flooding/salinization.

#### 5.2.1 Landscape: Non-rural landcover

Higher OWB within communities with no cropland (Model 2), could represent the greater availability of higher paid, non-primary livelihoods, such as construction and tourism within peri-urban landscapes (Sagoe-Addy and Addo, 2013; Delta Alliance, 2019). Less-vulnerable livelihoods could reduce seasonal losses, and provide the financial certainty required to increase actors’ confidence when making productive investments (Davis, 2014).

“[hospitality sector] is more of a sustainable job … but … farmers in the hinterlands only have … farming and fishing, so when rain comes to destroy their farms they are handicapped” (3,DPO)

However, despite financial benefits, non-rural households are associated with lower SWB (Model 2). Firstly, peri-urban landscapes and livelihoods could stimulate a money-orientated focus and greater individualism (Guillen-Royo and Velazco, 2012), contrasting the subjective benefits available from collectivism in agricultural communities.

“we are one people but things are getting difficult. Now we don’t have love among us anymore, we gather, talk and laugh, but when someone is going far then others are envious” (1,CI,M)

Greater visibility of investment and higher inequality within district capitals and peri-urban communities (Supplementary material S10), potentially driven by greater individualism and varying access to non-primary employment, can create greater relative comparability. Consequently, this may lower SWB if individuals’ higher aspirations are unmet (Kingdon and Knight, 2007; Kangmennaang et al., 2019). For example, new hospitality jobs in Sogakope (Site 3) were predominantly given to incoming Accra migrants, rather than locals.

“the same people that make us hungry are the same people who feed us … the moment you move forward, then … rich people invest to sell it at a low price so that ends your business” (3,CI,M)

However, individualism is not universal across non-rural locations, with strong place attachment shown to stimulate collective responses to climatic hazards (Leviston et al., 2018), even within communities with lower poverty levels.

“we are each other’s keeper so we ensure that the [flood] affected persons are … taken care of … I love the community, no matter how long I stay away I still come back” (8,FG,M)

Yet, even if climatic hazards stimulate community togetherness, and potentially increase SWB, certain coastal respondents highlighted how underlying motives can still be individualistic.

**If everyone is doing well, how do you feel?** …“You will feel good … because nobody will come and be a burden on you … If everyone is progressing then you will also be progressing” (8,CI,F)

Furthermore, multiple respondents noted “money is blood,” where money is “a constant humanizing flow essential to sustaining the physical, emotional, and social vitality of Ghanaian life” (Hasty, 2005, p. 276). Therefore, households with higher financial OWB would be expected to have stronger family and community ties, and potentially possess higher SWB (Meyer, 1998). However, recognizing these social bonds to only exist due to others’ individualistic monetary aspirations, caused unhappiness amongst some peri-urban respondents.

“When I was fully employed I usually get calls … from family because people know at the end of the month I will give them something, now I go a whole year without a single call” (1,FG,M)

Similar to agricultural participants, non-rural respondents also noted inconsistent community cohesiveness, suggesting social capital is fluid and capable of influencing OWB and SWB differently across time. For example, Anyamam (Site 2) participants recorded collective actions when responding to flood damage, with greater access to non-primary livelihoods and migration networks potentially increasing the capacity to provide monetary and material support. However, individualism appeared to prevail during everyday income-generating activities.

“if there is an accident we all come together and help … if we all have to contribute… we will do it” … **[yet, later stated]** … “when we go to the market together and they buy mine but they do not buy hers then that is it, the relationship has been destroyed … we are not looking for the wellbeing of others … instead of being happy for her, you carry hatred in your heart” (2,FG,F)

Despite individualism and monetary aspirations within non-rural locations potentially improving OWB, a desire for “quick money” could threaten communities’ SWB (Afriyie et al., 2016); producing “non-poor/unhappy” outcomes (Model 2). Fulfilling social expectations is crucial for a “good life” across Ghana (Dzokoto et al., 2019), therefore if “quick money” disturbs social norms, or threatens security, another key component of SWB (Vladisavljević and Mentus, 2019), community-wide happiness may be reduced. This concern was illustrated in Sogakope (Site 3) where monetary ambitions amongst younger community members, potentially linked to the capacity for financial capital to heighten social status (Ward and King, 2016), increased crime.

“[More money] is their target, they will do whatever they can to get there. We do not care if you are a thief, the moment you are rich everyone bows to you” (3,CI,M)

The goal of accumulating financial capital, perceived to be particularly pertinent amongst peri-urban youth, could also disincentivize productive, long-term human capital investments, such as education (Brown et al., 2021). Therefore, prioritizing monetary wealth potentially contributes towards “non-poor/unhappy” outcomes if self-development and generational improvements, key components of SWB within Ghana (Sulemana and Agyapong, 2019; Appiah et al., 2022), are sacrificed for short-term financial gains.

“with [salt] mining you get up and go to the shores, day ends you get something, they are valuing the money over going to school, so they do not have employable skills” (2,DPO)

Changing landscape characteristics are also hypothesized to challenge social norms and SWB. For example, increased land prices in Ningo Prampram district (Site 1), due to industrial expansion in west-Volta Delta (Doan and Oduro, 2012; Delta Alliance, 2019), encouraged self-gain amongst landlords at the expense of community wellbeing. This shift contested collective cultural expectations and the traditional “Ubuntu” worldview (Bangura, 2005), potentially producing societal concern and reducing community-wide SWB (Dzokoto, 2012).

“the landlords gave the land for free, whoever wants to farm on them … but now as the selling of land became lucrative … now they sell outright so even if you want to farm there is no land for you” (1,FG,M)

#### 5.2.2 Landscape: remoteness

Greater connectivity within peri-urban locations and district capitals may also improve OWB. For example, riverine vegetation, located around the South Tongu district capital, Sogakope (Site 3, Figure 3) increased the odds of being “non-poor/unhappy” (Model 2). Respondents noted how road access can stimulate growth and allow individuals to buy and sell where it is most profitable (Owusu and Lund, 2004; Chamberlin, 2007). These locations also often have lower institutional isolation, meaning OWB could also be improved through greater public investment into basic services, and private capital injections (Karakara and Ortsin, 2022).

“this place has become a central point, people come from different towns to this place even metro bus station is located here … This brought the building of hotels … and helped the construction of our main street” (6,FG,M)

For example, public investment into Senior High Schools or Universities may provide financial benefits to peri-urban communities, such as non-primary employment options (Morton, 2004) or increased adaptive capacity during unfavorable climates (Muttarak and Lutz, 2014). However, despite multiple respondents noting education to facilitate adaptation, no significant interaction effects were found (Model 2).

“where the world is going if you don’t have education you’re lost” …“without education you’ll be hooked on the manual way of farming” (7,FG,M)

Nonetheless, higher education access within peri-urban locations may enhance individuals’ aspirations (Anyidoho et al., 2012), which if unmet, potentially due to wider societal restraints, can cause frustrations (Graham and Pettinato, 2006; Szaboova et al., 2021). For example, one DPO mentioned how the systemic divide between Ghana’s education system and job market is increasing graduate unemployment, which can potentially lower community SWB due to the perceived “laziness” of unemployed youths disrupting social expectations (Grischow, 2008).

“now everyone wants to go to school … but if you complete school what do you get, you come back to where you were sitting” (1,FG,M)

Residing closer to major towns may also improve OWB by improving accessibility to migration networks and lubricating the transfer of information and labor (Awumbila et al., 2017). Communities with good connectivity may therefore objectively benefit from year-round, rather than seasonal, labor (Mortreux and Adams, 2015; Flahaux and De Haas, 2016). However, in contrast, persistent migratory pathways could lower SWB by increasing the visibility of higher living standards, and creating greater relative comparability; supporting the “non-poor/unhappy” outcome.

“Those who go to Tema and Accra … have more money than us … Because of where they are and the things they have their bodies are looking nice but those of us here we are not looking nice” (7,FG,F)

Yet, these broad ideas regarding inequality and SWB are not constant across non-rural locations. A respondent in Aflao (Site 6) stated “all fingers are not equal,” which was also referenced in rural Nyitawuta (Site 4). This phrase illustrates an “acceptance” of inequality within everyday life, possibly reducing its negative impact on SWB for certain individuals (Li et al., 2019).

“I just play my role in the best possible way … and pray that I also do well in my endeavors. All hands are not equal, so some will definitely do well than others” (2,CI,M)

#### 5.2.3 Landscape: proximity to water sources

Alongside less-vulnerable employment options within coastal/riverine peri-urban towns, these locations, and wetland landscapes (Model 2), may also objectively benefit from their proximity to water sources and other supporting/provisioning ecosystem services (Power, 2010). For example, access to rivers, lagoons and coastal aquifers (Nielsen et al., 2007) can improve soil fertility and accommodate irrigation farming (Warner et al., 2012; Mohammed et al., 2013), potentially providing a market advantage by facilitating year-round crop production.

“as we are able to do irrigation, the farmers are waiting for rains, so there is not as much of a supply, so when they hear we have okra they come rushing” (7,CI,M)

Higher, and more-consistent, financial OWB within coastal/riverine locations could also be attributed to diverse livelihood options (Burney and Naylor, 2012; Antwi-Agyei et al., 2014; Mahama and Nkegbe, 2021). For example, riverine farmers could “top-up” household incomes with seasonal fishing (Béné and Friend, 2011) or reed harvesting/mat weaving (Ayivor, 2014). Coastal communities, especially those with access to mangroves (Tanner et al., 2014), and both oceanic and freshwater (lagoon) resources, were also shown to exploit different locations across the year to maximize financial returns.

“when it comes to a critical point you can still visit the lagoon for fishing and even if the-farm does not do well you will definitely get something small from the lagoon for sale which will sustain you. Other communities do not have these options” (8,FG,M)

“they plant [mangroves] and leave it when they do their other activities like farming and fishing … when mangrove develops they harvest it and sell, but it is not their primary economic activity, it’s a top-up” (8,DPO)

However, despite OWB benefits, Model 2 illustrates coastal, riverine and wetland landscapes to potentially experience opposing low SWB. Taking wetlands as an example, these landscapes are ideal for cultivating subsistence crops, including rice (Willoughby et al., 2001), and profitable cash crops, such as sugarcane. Studies in Volta Delta show sugarcane farmers to achieve comparatively higher incomes and consumption, potentially linked to the emerging use of sugarcane as biofuel, and its lower sensitivity to flooding. Yet, Ahmed et al. (2019) depicted industrial sugarcane farmers in South Tongu district as “frustrated achievers.” Uncertain market access and fears of climate hazards, such as floods which previously caused crop failure, prevented these farmers from experiencing psychological, as well as financial, benefits.

Similarly, multiple coastal respondents noted how changing environmental conditions have contributed towards dwindling fish stocks and species extinction (Rahman and Schmidlin, 2014; Tanner et al., 2014; Koomson et al., 2020). Therefore, despite community observations suggesting fishing livelihoods were sufficient to prevent absolute poverty within select communities, lower harvests and trade incomes compared to past reference points could have still negatively impacted SWB amongst fishers *and* integrated tradespeople (Cazcarro et al., 2018); creating high OWB/low SWB outcomes (Model 2).

“In the past … we collected the harvest from the sea with so many basins but nowadays that is difficult” (6,FG,F)

“my business … they do not buy from me as much as I would like. When it is fishing season that is when they patronize my business, but nowadays the fishing season is not like it was in the past” (2,CI,F)

Reduced natural resource access was also specifically referenced in Anyamam (Site 2); with a comparatively high proportion of “non-poor/unhappy” households (Figure 3). Communities previously accessed year-round resources, with wet season fishing and dry season salt mining in Songor Lagoon (Roland et al., 2019). However, government ownership of salt resources (Parliament of the Republic of Ghana, 2006; Atta-Quayson, 2023), potentially linked to its use within the emerging petroleum industry (Mensah and Botchway, 2013), resulted in the entire 41,000 acre concession being sold to private investors (Harvey and Langdon, 2010; Yeboah et al., 2013). This example illustrates Ghana’s colonial, and post-colonial, extraction-based development pathway (Atta-Quayson, 2023). Communities were unhappy about their source of income and identity being removed, without compensation or alternative livelihoods being provided. Violent conflicts between resistant communities and privately funded police (Agbove, 2022) also reduced individuals’ perceived security; a core component of SWB (Vladisavljević and Mentus, 2019). This example illustrates why environment-wellbeing relationships should be explored within the “relational context” (White, 2010), as despite proximity to diverse resources having the capacity to improve SWB, the overarching governance structure reverses this assumed relationship.

“You don’t have the right to take any salt out of the lagoon and this has brought about war between town people and the individual … the war is intense … affecting our peace in the community” (2,FG,M)

#### 5.2.4 Climate hazards: flooding and salinization

The previously mentioned OWB benefits of non-rural landscapes, primarily located in coastal regions (Supplementary material S11), are contrasted by the capacity for climate hazards, including flooding and salinization (Model 2), to reduce OWB and/or SWB. This section focuses on negative SWB effects to explore potential processes behind “non-poor/unhappy” outcomes.

Firstly, flooding and salinization can increase health issues, including hypertension from consuming saline water (Nahian et al., 2018) and cholera; frequently referenced in communities which practice open-defecation (Dika, 2017). Ill-health may curtail OWB by lowering household productivity; however, even if not resulting in poverty, SWB can still be reduced, with “good health” a crucial determinant of SWB in LMICs (Van Praag et al., 2003; Addai et al., 2014; Osei-Tutu et al., 2020).

“as long as I am alive, and I am healthy, I am happy” (7,CI,M)

Climatic hazards may also exacerbate inequality, and consequently lower SWB, by increasing local relative comparability (Ravallion, 2014). For example, successful fishers in Anyamam (Site 2) invest in assets and properties within the nearby district capital due to fears of flooding along the coast, creating visible differences in living standards.

“most big houses in Sege [district capital] are owned by people in Anyamam. They use Anyamam as a business point and do their fishing … but in terms of assets they come and do it [in Sege] … they know that anytime the floods can take over” (2,DPO)

Next, drawing on eudaimonic SWB (Deci and Ryan, 2012), coastal flooding or intense rainfall can make roads and rivers impassable (Porter, 2007; Amankwaa and Gough, 2023), restricting autonomous mobility and potentially lowering SWB; particularly amongst communities accustomed to good connectivity. Flooding may also limit autonomy, and disrupt identities and social relationships, by preventing individuals from pursuing their desired livelihood and choosing where they live (Osei-Tutu et al., 2020; Babanawo et al., 2023).

“we are in the middle of water bodies, so when there is a flood, how do you go out to work? … some of us will lose our job” (1,FG,M)

“if your source of income is through fishing and now because of tidal waves you have been moved out of your land … you have to start all over again” (8,DPO)

Continuing discussions of community displacement, Model 2 highlights low community/place attachment to associate with “non-poor/unhappy” households. As discussed, low attachment, and an individualistic culture, can improve OWB by granting individuals’ greater control over their investments, and reducing obligations to share resources (Di Falco and Bulte, 2009).

“when people think individually they know the consequences of every decision … they are very analytical in making economic decisions … But if it is a group it is for nobody … that attachment … is not the same” (3,DPO)

However, low attachment can limit access to cultural ecosystem services, nature-based mental health benefits (Grinde and Patil, 2009; Heerwagen, 2009; Ingulli and Lindbloom, 2013; Wolsko and Lindberg, 2013) and higher levels of community togetherness, which are important components of SWB (Quinn and Halfacre, 2014; Ma et al., 2022; Schutte et al., 2022).

In contrast, qualitative findings suggest *high* attachment could exacerbate unhappiness within coastal communities if flooding results in involuntary displacement; opposing Model 2 which did not contain a significant interaction effect. Albrecht et al. (2007) developed “solastalgia”; “distress … produced by environmental change impacting on people while they are directly connected to their home environment” (p. S95). Therefore, displacement could lower SWB by challenging individuals’ identities and social relationships, often forged within communities’ traditional lands (Adams et al., 2020; Brown et al., 2021).

“That was their land, that was where they were born, so they do everything there, they have an attachment … they have been resettled, but some… still go back” (8,DPO)

Yet, place/community attachment was shown to fluctuate amongst coastal communities. Respondents noted how erosion, constant flooding, and reduced provisional ecosystem services (i.e., fish stock), which contribute towards social norms and practices (Kankam et al., 2021), weakened their emotional attachment to others and place, potentially lowering community-wide SWB.

“as the sea keeps on removing us, people have not relocated back. They don’t even visit here … because the sea always disturbs us” (7,FG,M)

Flooding and salinization may also generate low SWB (Model 2) by causing long-term psychological impacts. Fears of future devastation (Galway et al., 2019) were noted in all visited coastal communities, whereas Sekulova and Van den Bergh (2016) highlight how past unpreparedness can induce feelings of guilt, potentially disrupting the “calmness” needed for a “good life” (Osei-Tutu et al., 2020) and permanently lowering SWB.

“if you are unfortunate to have your work located at the shores of the sea you’ll always be in fear” (2,FG,M)

The potential for long-term SWB impacts could uncover why certain households continue to experience unhappiness, even during periods of relative objective prosperity. Furthermore, high OWB/low SWB could result from the capacity to detach OWB from environmental conditions, through irrigation for example, compared to the inability to detach subjective emotions from climatic hazards. “Non-poor/unhappy” outcomes may also arise due to policies targeting OWB being easier to implement than policies aiming to alter subjective perceptions. For example, providing building materials to recover from flood damage is more practical than boosting individuals’ multifaceted happiness (White, 2010).

Furthermore, communities’ powerlessness to combat climate hazards could dampen SWB by restricting individuals’ perceived autonomy, competence (mastery of environment) and capacity to care for nature; core components of eudaimonic SWB (Deci and Ryan, 2012; Navarro, 2017; Jax et al., 2018; Markussen et al., 2018). This negative effect could be intensified if communities are thought to be “politically isolated” (Sarracino, 2010; Asiedu et al., 2013) and possess low “linking” social capital; “relationships between people who are interacting across explicit, formal or institutionalized power or authority gradients “(Claridge, 2018, p. 4). This hypothesis was illustrated by Aflao’s (Site 6) DPO recalling how communities’ frustrations regarding government’s poor communication and limited progress towards constructing sustainable coastal defenses resulted in roadblock protests.

## 6 Conclusion

Using Volta Delta as a case study, this paper illustrated the potential for environmental conditions to influence OWB and SWB differently. A disparity was found between inland, agricultural and non-rural coastal/riverine locations. Agricultural communities associated with low OWB, likely influenced by their vulnerable livelihoods and remoteness from key services, yet higher SWB, potentially due to the focus on social relationships, and lower comparative reference points when self-defining happiness. In contrast, non-rural coastal/riverine communities associated with high OWB, possibly due to less-vulnerable employment options and greater public investment, yet lower SWB, likely due to higher inequality, unreachable money-orientated reference points, and psychological impacts from coastal hazards.

However, these broad generalizations were not homogenous across visited communities. For example, social capital was shown to be “fluid,” with the assumed collective/individualistic divide between rural/non-rural communities challenged in certain contexts. Agricultural respondents noted harmonious relationships, but acknowledged how collectivism is restricted during climatic hazards. Conversely, despite evidence of reciprocal relationships during coastal hazards, the persistence of broader, self-maintaining inequalities in non-rural locations also reinforced individualistic attitudes.

Overall, this study shows environmental conditions and wellbeing to be interlinked. It is recognised how this paper explores environment-wellbeing relationships as a snapshot, with future climatic changes potentially reshaping these associations. Nevertheless, using a mixed method approach to explore unaligned OWB/SWB outcomes highlights the need for sustainable development to contribute more towards understanding wellbeing measurements and conceptualizations at the local level. We recommend including OWB and SWB measures within sustainability policy to avoid negative trade-offs between desired outcomes, and to ensure wellbeing is comprehensively captured and targeted across different spaces with varying environmental conditions.

## Data availability statement

Publicly available datasets were analyzed in this study. This data can be found at: https://data.mendeley.com/datasets/223z53kwnm/1. The raw qualitative data supporting the conclusions of this article will be made available by the authors, without undue reservation.

## Ethics statement

The studies involving humans were approved by University of Southampton ERGO II (Faculty of Environmental and Life Sciences (FELS)) Case number: 77828. The studies were conducted in accordance with the local legislation and institutional requirements. The participants provided their written informed consent to participate in this study. Written informed consent was obtained from the individual(s) for the publication of any potentially identifiable images or data included in this article.

## Author contributions

LC: Writing – review & editing, Writing – original draft, Visualization, Project administration, Methodology, Investigation, Funding acquisition, Formal analysis, Data curation, Conceptualization. CH: Writing – review & editing, Supervision. AS: Writing – review & editing, Supervision. KN: Writing – review & editing, Supervision.

## Footnotes

^1^Vulnerability defined by the Development Assistance Research Associates (DARA, 2012) metric which includes “22 indicators across four Impact Areas (Environmental Disasters, Habitat Change, Health Impact, Industry Stress).

^2^Enumeration areas were selected proportional-to-size, and are designed to have similar household counts (*n* = 150) (Cordes et al., 2021), therefore it was assumed households have approximately equal selection probabilities (Groves et al., 2011).

^3^2 km buffers were drawn around each household in an enumeration area then merged together (using the ArcGIS *dissolve* tool).

^4^Inland water distance was obtained from the European Space Agency CCI (Climate Change Initiative) LC (Land Cover project) (Lamarche et al., 2017). Data on proximity to major roads and intersects was taken from OpenStreetMap.

^5^Based on a car travelling along recognized road networks as at December 3rd, 2021, at 8:00 am.

^6^Note, due to missing data for select questions, 1,360 households had a valid expenditure poverty classification, whereas 1,364 had a valid unhappiness outcome. Only households with results for both measures were included in the combined dependent variables.

^7^Only one semi-structured interview was carried out in Site 1 (Afienya) due to time restraints and two potential participants dropping out early in the interview process.

^8^Spoken consent was received for the two telephone interviews.

^9^Verbatim quotes will be classified by the site number (Table 2), community focus group/community interview/district planner interview (FG/CI/DPO), and gender (M/F). For example, a female focus group in Afienya would be denoted by (1,FG,F).
