# Supplementary material for: Opposing objective and subjective wellbeing outcomes within an environmentally vulnerable delta: a case study of Volta Delta, Ghana
Source: Front Psychol. 2024 Aug 23;15:1401480. doi: 10.3389/fpsyg.2024.1401480 (PMC11377353; doi:10.3389/fpsyg.2024.1401480)
Supplement: Supplementary file 1 [file Data_Sheet_1.docx]

Supplementary Material

Opposing objective and subjective wellbeing outcomes within an environmentally vulnerable delta: a case study of Volta Delta, Ghana

**Supplementary material S1**

Variables tested within the regression models, categorised by environmental and control characteristics. Note, some variables have different categorisations to ensure a minimum cell size is maintained across the two outcomes. Also, numerical values were categorised if the numerical variable violated the assumption of linearity with the log odds of the dependent variable. Information on how categorisations were defined is also presented.

| **Variable** | **Coding** | **Data source** |
| --- | --- | --- |
| **Environmental variables** |  |  |
| **Climate** | |  |
| **Climate shocks** |  |  |
| Exposure to storms/flooding | 0: Not exposed to storms/flooding  1: Exposed to storms/flooding | DECCMA |
| Environmental impact from storms/flooding | 0: Not environmentally impacted by storms/flooding  1: Environmentally impacted by storms/flooding | DECCMA |
| Economic impact from storms/flooding | 0: Not economically impacted by storms/flooding  1: Economically impacted by storms/flooding | DECCMA |
| **Climate stresses** |  |  |
| Exposure to drought/salinity/ erosion | 0: Not exposed to drought/salinity/ erosion  1: Exposed to drought/salinity/ erosion | DECCMA |
| Environmental impact from drought/salinity/erosion | 0: Not environmentally impacted by drought/salinity/erosion  1: Environmentally impacted by drought/salinity/erosion | DECCMA |
| **Variable** | **Coding** | **Data source** |
| Economic impact from drought/  salinity/erosion | 0: Not economically impacted by drought/salinity/erosion  1: Economically impacted by drought/salinity/erosion | DECCMA |
| **Climate shifts/seasonality** |  |  |
| Change in rainfall over the last 5 years | 0: Stayed the same 1: Changed (increased/decreased/changed another way) | DECCMA |
| Change in rainfall timing over the last 5 years | 0: Stayed the same 1: Earlier rainy season 2: Later rainy season | DECCMA |
| Change in temperature over the last 5 years | 0: Stayed the same 1: Changed (increased/decreased/changed another way) | DECCMA |
| **Landscape** | |  |
| **Remoteness** |  |  |
| Distance to coast | 0: 0-<10km  1: 10-20km  2: 20km+  *(10km increments)* | WorldPop (US NASA Shuttle Radar Topography Mission (SRTM)) |
| Distance to inland water (km) | Numeric (km) | WorldPop (European Space Agency CCI) |
| Travel time to Accra | 0: 1-2 hours  1: 2-3 hours  2: 3+ hours  *(1 hour increments)* | Produced using Google API data |
| Travel time to district capital | 0: 0-10 mins  1: 11-20mins  2: 21-30 mins  3: 30+ mins (reference)  *(10-min increments)* | Produced using Google API data |
| Distance to major road | 0: 0-1.77km  1: 1.78-3.93km  2: 3.93km+  *(natural breaks)* | WorldPop (OpenStreetMap) |
| Distance to road intersect | 0: 1-3.15km  1: 3.16-6.3km  2: 6.3km+  *(natural breaks)* | WorldPop (OpenStreetMap) |
| Region | 0: Volta 1: Greater Accra | x |
| **Variable** | **Coding** | **Data source** |
| **Topography** |  |  |
| Elevation | 0: 0-10.5masl  1: 10.6-27masl  2: 27masl +  *(natural breaks)* | WorldPop (US NASA Shuttle Radar Topography Mission (SRTM)) |
| Slope | 0: Under 1°  1: 1° or more  *(logical threshold)* | WorldPop (US NASA Shuttle Radar Topography Mission (SRTM)) |
| **Landcover** |  |  |
| EVI vegetation index (2016) | 0: 0.113-0.198  1: 0.199-0.269  2: 0.270+  *(natural breaks)* | NASA (MODIS) |
| EVI % change (2006-16) | 0: High decrease (-50% to -15%)  1: Low decrease (-14% to 0%)  2: Low increase (0% to 17%)  3: High increase (18% to 52%)  *(two natural breaks above/below 0%)* | NASA (MODIS) |
| EVI % change (2011-16) | 0: High decrease (-55% to -19%) 1: Low decrease (-19% to 0%) 2: Low increase (0% to 25%) 3: High increase (26% to 63%)  *(two natural breaks above/below 0%)* | NASA (MODIS) |
| EVI % change (2015-16) | 0: High decrease: (-57% to -36%) 1: Medium decrease: (-35% to -19%) 2: Low increase: (-18% to 0%) 3: Increase: (Over +0%)  *(natural breaks below 0%)* | NASA (MODIS) |
| Cropland | 0: 0% (None)  1: 0-25% (Low)  2: 26-55% (Medium)  3: 55%+ (High)  *(0% group, and natural breaks)* | LANDSAT-7, FAO Classifications |
| Savannah grassland | 0: 0%  1: 0-20%  2: 20+%  *(0% group, and natural breaks)* | LANDSAT-7, FAO Classifications |
| **Variable** | **Coding** | **Data source** |
| Riverine vegetation | 0: Riverine vegetation not present in community buffer  1: Riverine vegetation present in community buffer | LANDSAT-7, FAO Classifications |
| Mangrove | 0: Mangrove not present in community buffer  1: Mangrove present in community buffer | LANDSAT-7, FAO Classifications |
| Wetland | 0: Wetland not present in community buffer  1: Wetland present in community buffer | LANDSAT-7, FAO Classifications |
| Bare land | 0: Bare land not present in community buffer  1: Bare land present in community buffer | LANDSAT-7, FAO Classifications |
| Marshland | 0: Marshland not present in community buffer  1: Marshland present in community buffer | LANDSAT-7, FAO Classifications |
| Lagoon | 0: Lagoon not present in community buffer  1: Lagoon present in community buffer | LANDSAT-7, FAO Classifications |
| Beach | 0: Beach not present in community buffer  1: Beach present in community buffer | LANDSAT-7, FAO Classifications |
| Salt pan | 0: Salt pan not present in community buffer  1: Salt pan present in community buffer | LANDSAT-7, FAO Classifications |
| Built-up land | 0: 0% - 16% (None/low)  1: 17% - 38% (Medium)  2: >39% (High)  *(natural breaks)* | LANDSAT-7, FAO Classifications |
| % change in built-up land (2001-15) | 0: No change (-1% > < +1%)  1: Decrease (<-1%)  2: Increase (>+1)  *(logical groups)* | LANDSAT-7, FAO Classifications |
| **Control variables** |  |  |
| **Household characteristics** |  |  |
| Household size | *5-group categorisation (logical groups)*  1: 1 person  2: 2-3 people  3: 4-5 people  4: 6-7 people  5: 8+ people  *2-group categorisation (above/below median)*  1: 1-4 people  2: 5+ people | DECCMA |
| **Variable** | **Coding** | **Data source** |
| Livelihood cluster | 0: Salaried employee/business owner  1: Fishing/trade/transport/construction  2: Crop farmer  *(k-means cluster analysis)* | DECCMA |
| Dependants in households | 0: 0  1: 1 to 2  2: 3 to 4  3: 5+  *(logical groups)* | DECCMA |
| Child:adult dependency ratio | *Numeric value*  Child/adult ratio | DECCMA |
| How long lived in the village? | 0: Entire life  1: Migrated more than 10 years ago  2: Migrated less than 10 years ago | DECCMA |
| Proportion of females | *Numeric value*  % females in household | DECCMA |
| Religion | 0: Christian  1: Non-Christian (Islam, Buddhism, Hinduism, Traditional) | DECCMA |
| **Household head characteristics** |  |  |
| Years of schooling | 0: No Schooling  1: Below-basic education  2: Basic education  3: Above-basic education | DECCMA |
| Marital status | 0: Married or cohabitating  1: Never married  2: Previously married | DECCMA |
| Employment status | 0: Permanent  1: Non-permanent  2: Dependent (unemployed, student or retired) | DECCMA |
| Sex | 0: Male  1: Female | DECCMA |
| Age | 0: 12-33 years  1: 34-45 years  2: 46-60 years  3: 60+ years  *(quartiles)* | DECCMA |
| **Adaptation** |  |  |
| Any form of adaptation (last 5 years) | 0: No adaptation in the last 5 years  1: At least one form of adaptation in the last 5 years | DECCMA |
| Current migrant outside household | 0: No migrant currently outside household  1: Migrant currently outside household | DECCMA |
| **Variable** | **Coding** | **Data source** |
| Past migrant outside household | 0: No migrant previously outside household  1: Migrant previously outside household | DECCMA |
| Intention to migrate? | 0: No intention  1: Intention to migrate | DECCMA |
| Migration evaluation | 0: Not helpful/neither helpful nor unhelpful  1: Helpful | DECCMA |
| **Assets** |  |  |
| Latrine type | 0: Flushing latrine  1: Pit latrine, Public latrine or KVIP  2: No facility | DECCMA |
| Drinking water source | 0: Piped/tubewell/standpipe  1: Dug well/open source | DECCMA |
| Roof material | 0: Non-secure  1: Secure | DECCMA |
| Home ownership | 0: Not owned (mortgaged, renting, squatting)  1: Owned | DECCMA |
| **Subjective evaluations** |  |  |
| Place/community attachment | 0: Low  1: Medium  2: High  *(PCA & cluster analysis)* | DECCMA |
| Personality | 0: Low  1: Medium  2: High  *(PCA & cluster analysis)* | DECCMA |
| **Community characteristics** |  |  |
| Population density (2016) | 0: Low (74 - 369 people/km^2^)  1: Medium (370 - 877 people/km^2^)  2: High (878 - 2702 people/km^2^)  *(natural breaks)* | WorldPop |

**Supplementary material S2**

Information used to calculate absolute, equivalised, adjusted expenditure poverty. (S2.1) Expenditure data types included (S2.2) Regional cost-of-living indices (S2.3) GSS calorie-based household equivalisation scale.

**(S2.1)** *Expenditure types included in the calculation of the expenditure poverty measure. The mean percentage of household expenditure for each type is also reported.*

| **Expenditure type** | **Mean % annual expenditure** |
| --- | --- |
| Food | 59% |
| Household essentials (inc. electricity & transport) | 12% |
| Education | 9% |
| Health | 4% |
| Livelihood inputs (inc. fertilisers & equipment) | 4% |
| Sporadic house costs (inc. home improvements & repairs) | 2% |
| Non-essential household costs (inc. furniture & clothing) | 1% |
| “Other” (inc. funerals & weddings) | 1% |
| Insurance | <1% |
| Supporting migrants in hardship | <1% |

**(S2.2)** *Indices are produced over the 12-month period of the Ghanaian Living Standards Survey No. 7 (2017) (produced with Greater Accra January 2017 as the reference point). Note, the indices for a region are calculated as the monthly average over a 12-month period; therefore explaining why the index for Greater Accra is not 1.0, despite being the reference location (GSS, 2018).*

| **Region** | **Overall price index** | **Food index** | **Non-Food index** |
| --- | --- | --- | --- |
| Western | 1.02 | 1.00 | 1.04 |
| Central | 0.98 | 0.94 | 1.03 |
| **Greater Accra** | **1.03** | **1.02** | **1.03** |
| **Volta** | **0.99** | **0.93** | **1.07** |
| Eastern | 0.95 | 0.94 | 0.96 |
| Ashanti | 0.96 | 0.90 | 1.03 |
| Brong Ahafo | 0.93 | 0.91 | 0.97 |
| Northern | 0.97 | 0.98 | 0.97 |
| Upper East | 0.86 | 0.80 | 0.93 |
| Upper West | 0.92 | 0.90 | 0.96 |

**(S2.3)** *Equivalation scale used by the GSS, based on dietary requirements of different ages and sexes - recorded by the National Research Council in 1989 (GSS, 2018).*

| **Category** | **Age group (years)** | **Average energy allowance per day (kcal)** | | **Equivalence scale** |
| --- | --- | --- | --- | --- |
| **Infants** | <1 | | 650 | 0.22 |
| **Children** | 1 to 3 | | 1,300 | 0.45 |
|  | 4 to 6 | | 1,800 | 0.62 |
|  | 7 to 10 | | 2,000 | 0.69 |
| **Adult males** | 11 to 14 | | 2,500 | 0.86 |
|  | 15 to 18 | | 3,000 | 1.03 |
|  | 19 to 25 | | 2,900 | 1.00 |
|  | 26 to 50 | | 2,900 | 1.00 |
|  | 51+ | | 2,300 | 0.79 |
| **Adult females** | 11 to 14 | | 2,200 | 0.76 |
|  | 15 to 18 | | 2,200 | 0.76 |
|  | 19 to 25 | | 2,200 | 0.76 |
|  | 26 to 50 | | 2,200 | 0.76 |
|  | 51+ | | 1,900 | 0.66 |

**Supplementary material S3**

Crosstabulation and chi-square test of association between general (global) happiness and happiness with community interactions (Table S3a) and economic security (Table S3b). The 5-point Likert scale was aggregated into a 3-point scale, with moderately/very unhappy, and moderately/very happy combined.

| **Table S3a** | **Happiness with community interactions** | | |  |
| --- | --- | --- | --- | --- |
| **Happiness in general** | Happy | Neutral | Unhappy | Total |
| Happy | 830 (90%) | 50 (5%) | 43 (5%) | 923 |
| Neutral | 94 (78%) | 21 (17%) | 6 (5%) | 121 |
| Unhappy | 251 (78%) | 27 (8%) | 42 (13%) | 320 |
| **Total** | **1,175**  **(86%)** | **98**  **(7%)** | **91**  **(7%)** | **1,364** |
| **Chi-square statistic** | **52.989***** | | | |
|  |  |  |  |  |
|  |  |  |  |  |
|  |  |  |  |  |
| **Table S3b** | **Happiness with economic security** | | |  |
| **Happiness in general** | Happy | Neutral | Unhappy | Total |
| Happy | 471 (51%) | 132 (14%) | 320 (35%) | 923 |
| Neutral | 30 (25%) | 23 (19%) | 68 (56%) | 121 |
| Unhappy | 36 (11%) | 18 (6%) | 266 (83%) | 320 |
| **Total** | **537**  **(39%)** | **173**  **(13%)** | **654**  **(48%)** | **1,364** |
| **Chi-square statistic** | **239.208***** | | | |

**Supplementary material S4**

PCA loadings for individual domains in the life domains happiness index. All positively correlate with the first component.

| **Life Domain** | **First Principal Component loading** |
| --- | --- |
| Happiness with food security | +0.658 |
| Happiness with housing | +0.654 |
| Happiness with economic security | +0.640 |
| Happiness with family interactions | +0.637 |
| Happiness with environment | +0.631 |
| Happiness with community interactions | +0.621 |
| Happiness with health | +0.576 |
| Happiness with drinking water | +0.423 |

**Supplementary material S5**

Details of focus group and interview participants from all eight study sites. Age profiles for all community interviews, focus group members, and DPOs also presented. Note, some individuals did not disclose their age, also some individuals who consented left during FGs. However, as it is not possible to know which individual departed, their ages are included in the figures.

| **1) Afienya** | Male FG | Female FG | Community interviews | DPO interview |
| --- | --- | --- | --- | --- |
| No. participants | 7 | 6 | 1 (M) | 1 (F) |
| Duration | 01:45 | 01:38 | 00:33 | 00:55 |
| Age range | 32 - 49 | 21 - 55 | 32 – 41 | 42 - 51 |
| Language | English | Dangbe | Dangbe | English |
| Consent | Written | Written | Written | Written |

| **2) Anyamam** | Male FG | Female FG | Community interviews | DPO interview |
| --- | --- | --- | --- | --- |
| No. participants | 8 | 8 | 1 (M), 1 (F) | 1 (F) |
| Duration | 01:47 | 02:09 | 00:42 (M), 00:49 (F) | 00:59 |
| Age range | 28 - 48 | 25 - 62 | 32 – 41 (M & F) | 32 - 41 |
| Language | Dangbe | Dangbe | Dangbe | English |
| Consent | Written | Written | Written | Written |

| **3) Sogakope** | Male FG | Female FG* | Community interviews | DPO interview |
| --- | --- | --- | --- | --- |
| No. participants | 6 | 10 | 1 (M), 1 (F) | 1 (M) |
| Duration | 01:32 | 01:49 | 00:32 (M), 00:32 (F) | 00:46 |
| Age range | 30 - 57 | 34 - 55 | 26 – 31 (M), 18 – 25 (F) | 32 - 41 |
| Language | Ewe | Ewe | English (M & F) | English |
| Consent | Written | Written | Written | Written |

** Female FG respondents became quite aggressive, many were demanding money, or telling others to stop sharing their opinions.*

| **4) Nyitawuta** | Male FG | Female FG | Community interviews | DPO interview |
| --- | --- | --- | --- | --- |
| No. participants | 8 | 8 | 1 (M), 1 (F) | 1 (M) |
| Duration | 02:08 | 01:42 | 00:46 (M), 00:47 (F) | 00:36 |
| Age range | 20 - 80 | 36 - 63 | x (M), 42 – 51 (F) | x |
| Language | Ewe | Ewe | Ewe | English |
| Consent | Written | Written | Written | Spoken |

| **5) Awlikope** | Male FG | Female FG | Community interviews | DPO interview |
| --- | --- | --- | --- | --- |
| No. participants | 8 | 8 | 1 (M), 1 (F) | 1 (M) |
| Duration | 01:51 | 01:52 | 00:58 (M), 01:03 (F) | 00:41 |
| Age range | 35 - 50 | 26 - 62 | 62 – 71 (M), 32 – 41 (F) | 42 - 51 |
| Language | Ewe | Ewe | English (M), Ewe (F) | English |
| Consent | Written | Written | Written | Written |

| **6) Aflao** | Male FG* | Female FG | Community interviews | DPO interview |
| --- | --- | --- | --- | --- |
| No. participants | 7 | 8 | 1 (M), 1 (F) | 1 (M) |
| Duration | 01:04 | 01:27 | 00:37 (M), 00:39 (F) | 00:47 |
| Age range | 26 - 60 | 19 - 63 | 32 – 41 (M), 62 – 71 (F) | x |
| Language | Ewe | Ewe | Ewe (M), Ewe/Dangbe (F) | English |
| Consent | Written | Written | Written | Spoken |

** Male FG respondents were reluctant to stay and not very responsive.*

| **7) Kedzi** | Male FG | Female FG | Community interviews | DPO interview |
| --- | --- | --- | --- | --- |
| No. participants | 9 | 7 | 2 (M) | 1 (M) |
| Duration | 01:51 | 01:45 | 00:45, 01:02 (M) | 00:38 |
| Age range | 32 - 66 | 38 - 58 | 32 – 41, 42 – 51 (M) | 52 - 61 |
| Language | Ewe | Ewe | 2 English (M) | English |
| Consent | Written | Written | Written | Written |

| **8) Anloga** | Male FG | Female FG | Community interviews | DPO interview |
| --- | --- | --- | --- | --- |
| No. participants | 10 | 8 | 1 (M), 1 (F) | 1 (M) |
| Duration | 01:30 | 01:40 | 00:48 (M), 00:37 (F) | 00:46 |
| Age range | 28 - 63 | 18 - 59 | 32 – 41 (M & F) | 32 – 41 |
| Language | Ewe | Ewe | English (M), Ewe (F) | English |
| Consent | Written | Written | Written | Written |


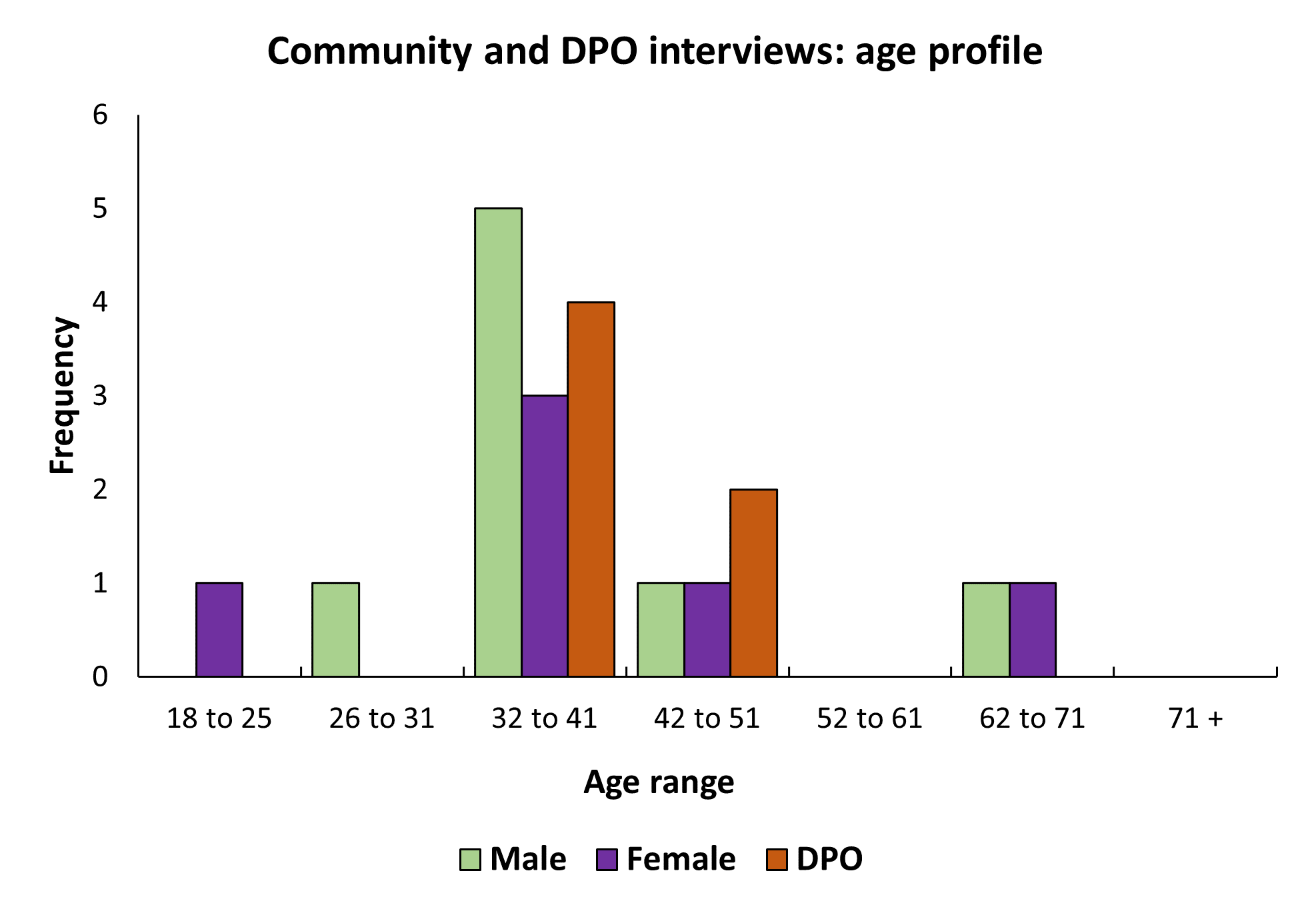

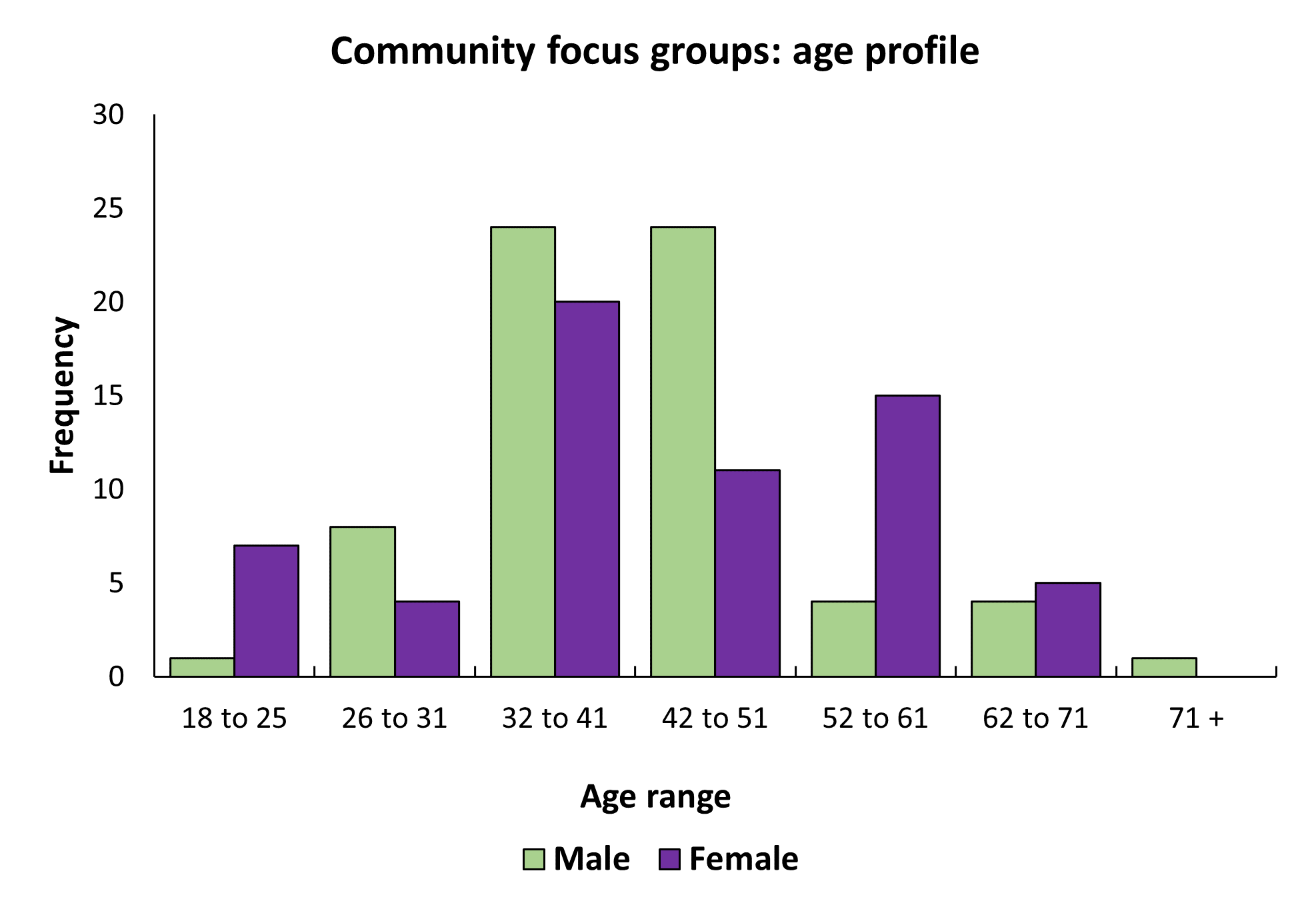


**Supplementary material S6**

Site-specific focus group question guide. Not all questions were asked, many were used as prompts if respondents needed encouragement. Environmental characteristics were based on remote sensing data, and where self-reported climatic hazards exceeded the DECCMA sample average:

Interview guide, with female-specific questions:

**Supplementary Material S7**

Descriptive statistics of the explanatory variables incorporated within the two binary logistic regression models^[[1]](#footnote-1)^.

| **Model** | **Variable group** | **Variable** | **Descriptive statistics** |
| --- | --- | --- | --- |
| **Model 1 – “poor/happy”** | Climate shocks & stresses | Economic impact from drought | Not impacted – 854 (63%)  Impacted – 440 (32%)  *Missing – 70 (5%)* |
|  |  | Economic impact from flooding | Not impacted – 1,105 (81%)  Impacted – 228 (17%)  *Missing – 31 (2%)* |
|  |  | Environmental impact from erosion | Not impacted – 886 (65%)  Impacted – 415 (30%)  *Missing – 63 (5%)* |
|  | Landscape/  remoteness | Region | Greater Accra – 486 (36%)  Volta – 878 (64%) |
|  |  | Cropland coverage | None – 227 (17%)  Low – 364 (27%)  Medium – 244 (18%)  High – 529 (39%) |
|  |  | River vegetation coverage | No coverage in community – 1,282 (94%)  Present in community – 82 (6%) |
|  | Physical assets | Drinking water source | Piped or tubewell – 1,060 (78%)  Dug well or open source – 304 (22%) |
|  |  | Latrine facility | Flushing – 107 (8%)  Pit/Public/KVIP – 711 (52%)  No facility – 546 (40%) |
|  | Household characteristics | Household size | 1 person – 242 (18%)  2-3 people – 401 (29%)  4-5 people – 409 (30%)  6-7 people – 204 (15%)  8+ people – 108 (8%) |
|  | Household head characteristics | Highest education level | None – 382 (28%)  Primary – 391 (29%)  Lower secondary – 338 (25%)  Higher secondary or above – 249 (18%)  *Missing – 4 (<1%)* |
|  | Adaptation | Current migrant out of household | No – 761 (56%)  Yes – 603 (44%) |

| **Model** | **Variable group** | **Variable** | **Descriptive statistics** |
| --- | --- | --- | --- |
| **Model 2 – “non-poor/unhappy”** | Climate shocks & stresses | Environmental impact from salinity | Not impacted – 921 (68%)  Impacted – 376 (28%)  *Missing 67 (5%)* |
|  |  | Exposure to flooding | Not exposed – 889 (65%)  Exposed – 444 (33%)  *Missing – 31 (2%)* |
|  | Landscape/  remoteness | Cropland coverage | None – 227 (17%)  Low – 364 (27%)  Medium – 244 (18%)  High – 529 (39%) |
|  |  | River vegetation coverage | No coverage in community – 1,282 (94%)  Present in community – 82 (6%) |
|  |  | Wetland coverage | No coverage in community – 407 (30%)  Present in community – 957 (70%) |
|  | Subjective characteristics | Place/community attachment | Low – 195 (14%)  Medium – 522 (38%)  High – 647 (47%) |
|  | Household characteristics | Household size | 1-4 person – 853 (63%)  5+ person – 511 (37%) |

**Supplementary material S8**

Methodological information on the construction of the “place/community attachment” variable.

The “place/community attachment” variable was constructed using ordinal Principal Components Analysis (PCA) (*princals R-function)* and cluster analysis. The ordinal components were measured on 5-point Likert scales (see below). The first dimension of the PCA analysis was extracted as the index value. Three clusters were consequently constructed to provide low/medium/high values, and to ensure sufficient sample size (>100) within each group.

**How well do the following statements describe you?: Disagree strongly → Agree strongly**

- The village is part of my life
- I want my family and friends to live here in the future
- I feel like an outsider in this place (scale reversed)
- I live here because it is practical
- I miss the place when I am not here
- My friends and family are a good support for me
- I enjoy being involved in village activities

**Supplementary material S9**

Chi-square analysis showing a significant overrepresentation of longer travel times to district capitals (top) and greater distance from major roads (bottom) amongst communities with high cropland coverage.

|  | **Cropland coverage in the community** | | | | |
| --- | --- | --- | --- | --- | --- |
| **Travel time to district capital** | **None**  **(0%)** | **Low**  **(1 – 25%)** | **Medium**  **(26 – 54%)** | **High**  **(55+ %)** | **Total** |
| 0 – 10 mins | 112 (28%) | 194 (48%) | 25 (6%) | 75 (18%) | 406 |
| 11 – 20 mins | 85 (25%) | 0 (0%) | 111 (33%) | 140 (42%) | 336 |
| 21 – 30 mins | 0 (0%) | 89 (34%) | 58 (22%) | 113 (46%) | 260 |
| 30+ mins | 30 (8%) | 81 (22%) | 50 (14%) | 201 (56%) | 362 |
| **Total** | **227**  **(17%)** | **364**  **(27%)** | **244**  **(18%)** | **529**  **(39%)** | **1,364** |
| **Chi-square test statistic** | **420.306***** | | | | |
|  | | | | | |
| **Distance to major road** | **None**  **(0%)** | **Low**  **(1 – 25%)** | **Medium**  **(26 – 54%)** | **High**  **(55+ %)** | **Total** |
| Low  (<1.5km) | 227 (23%) | 252 (25%) | 189 (19%) | 336 (33%) | **1,004** |
| Medium  (1.6 – 4km) | 0 (0%) | 83 (43%) | 55 (28%) | 57 (29%) | **195** |
| High  (>4km) | 0 (0%) | 29 (18%) | 0 (0%) | 136 (82%) | **165** |
| **Total** | **227**  **(17%)** | **364**  **(27%)** | **244**  **(18%)** | **529**  **(39%)** | **1,364** |
| **Chi-square test statistic** | **240.439***** | | | | |

**Supplementary material S10**

Chi-square analysis showing a significant overrepresentation of high community inequality (Gini coefficient) in highly built-up communities, and an overrepresentation of low inequality within more-rural communities.

|  | **Built-up landcover in community** | | | | |
| --- | --- | --- | --- | --- | --- |
| **Enumeration area expenditure inequality**  **(Gini coefficient)** | **None/low coverage**  **(0% - 8%)** | **Low-medium coverage**  **(9% - 19%)** | **Medium-high coverage**  **(20% - 44%)** | **High coverage (45% - 70%)** | **Total** |
| Low inequality  (0.226 – 0.320) | 233 (54%) | 87 (13%) | 57 (13%) | 51 (12%) | 428 |
| Medium inequality  (0.321 – 0.405) | 227 (43%) | 162 (31%) | 84 (16%) | 53 (10%) | 526 |
| High inequality  (0.406 – 0.536) | 192 (47%) | 79 (19%) | 53 (13%) | 86 (21%) | 410 |
| **Total** | **652**  **(48%)** | **328**  **(24%)** | **194**  **(14%)** | **190**  **(14%)** | **1,364** |
| **Chi-square test statistic** | **46.053***** | | | | |

**Supplementary material S11**

Chi-square analysis showing a significant overrepresentation of households closer to the coast when the community contains a larger proportion of built-up landcover.

|  | **Distance to coast (km)** | | |  |
| --- | --- | --- | --- | --- |
| **Built-up landcover in community buffer** | **0 – 10km** | **11 – 20km** | **>20km** | **Total** |
| None/low coverage  (0 – 16%) | 544 (59%) | 137 (15%) | 241 (26%) | 922 |
| Medium coverage  (17 – 38%) | 199 (89%) | 25 (11%) | 0 (0%) | 224 |
| High coverage (>38%) | 195 (89%) | 23 (11%) | 0 (0%) | 218 |
| **Total** | **938**  **(69%)** | **185**  **(14%)** | **241**  **(18%)** | **1,364** |
| Chi-square test statistic | **158.556***** | | | |

1. Note, two different categorisations for the “household size” variable are presented due to the need for adequate cell size (n>10) within the two models. [↑](#footnote-ref-1)
